# Identification of homologs of the *Chlamydia trachomatis* effector CteG reveals a family of Chlamydiaceae type III secreted proteins that can be delivered into host cells

**DOI:** 10.1007/s00430-024-00798-9

**Published:** 2024-07-15

**Authors:** Inês Serrano Pereira, Maria da Cunha, Inês Pacheco Leal, Maria Pequito Luís, Paula Gonçalves, Carla Gonçalves, Luís Jaime Mota

**Affiliations:** 1 Associate Laboratory i4HB - Institute for Health and Bioeconomy, NOVA School of Science and Technology, NOVA University Lisbon, Caparica, Portugal; 2 UCIBIO – Applied Molecular Biosciences Unit, Department of Life Sciences, NOVA School of Science and Technology, NOVA University Lisbon, Caparica, Portugal

**Keywords:** Bacterial pathogenesis, Chlamydia, Type III secretion, Phylogeny, Effector evolution

## Abstract

Chlamydiae are a large group of obligate endosymbionts of eukaryotes that includes the Chlamydiaceae family, comprising several animal pathogens. Among Chlamydiaceae, *Chlamydia trachomatis* causes widespread ocular and urogenital infections in humans. Like many bacterial pathogens, all Chlamydiae manipulate host cells by injecting them with type III secretion effector proteins. We previously characterized the *C. trachomatis* effector CteG, which localizes at the host cell Golgi and plasma membrane during distinct phases of the chlamydial infectious cycle. Here, we show that CteG is a Chlamydiaceae-specific effector with over 60 homologs phylogenetically categorized into two distinct clades (CteG I and CteG II) and exhibiting several inparalogs and outparalogs. Notably, *cteG* I homologs are syntenic to *C. trachomatis cteG*, whereas *cteG* II homologs are syntenic among themselves but not with *C. trachomatis cteG*. This indicates a complex evolution of *cteG* homologs, which is unique among *C. trachomatis* effectors, marked by numerous events of gene duplication and loss. Despite relatively modest sequence conservation, nearly all tested CteG I and CteG II proteins were identified as type III secretion substrates using *Yersinia* as a heterologous bacterial host. Moreover, most of the type III secreted CteG I and CteG II homologs were delivered by *C. trachomatis* into host cells, where they localized at the Golgi region and cell periphery. Overall, this provided insights into the evolution of bacterial effectors and revealed a Chlamydiaceae family of type III secreted proteins that underwent substantial divergence during evolution while conserving the capacity to localize at specific host cell compartments.

**Supplementary Information:**

The online version contains supplementary material available at 10.1007/s00430-024-00798-9.

## Introduction

Chlamydiae is a bacterial phylum containing obligate endosymbionts of eukaryotes, including unicellular protozoa and diverse animals [1, 2]. It contains species that have been isolated from various environments, the so-called environmental chlamydiae, which can be phylogenetically grouped into eight distinct families [2, 3]. In addition, the Chlamydiaceae family comprises the *Chlamydia* genus that includes human pathogens (*C. trachomatis* and *C. pneumoniae*) and several other established or proposed species that are pathogens of diverse animals and in some cases have been shown to have zoonotic potential [4]. Recently, among the Chlamydiaceae, the *Chlamydiifrater* genus was described, comprising two species isolated from flamingos [5]. Among Chlamydiae, *C. trachomatis* is the most studied species as it is a model obligate intracellular bacterial pathogen and causes trachoma and sexually transmitted diseases that affect millions of people worldwide [6].

Chlamydiae share a characteristic developmental cycle including the infectious, but non-replicative, elementary bodies (EBs) and the non-infectious, but replicative, reticulate bodies (RBs) [6, 7]. EBs invade host cells while forming a membrane-bound vacuolar compartment. Within this compartment, EBs differentiate into RBs, which multiply leading to the formation of a large vacuole, known as the inclusion. Eventually, RBs re-differentiate back into EBs. The cycle is completed by host cell egress of the infectious EBs, either by extrusion of the entire inclusion or by host cell lysis.

Another unifying feature of Chlamydiae is that they utilize a type III secretion (T3S) system to deliver effector proteins into host cells [6, 8–12]. Collectively, Chlamydiae effectors act on a wide range of eukaryotic cell processes at different stages of the developmental cycle to promote host cell invasion, immune evasion, chlamydial survival, proliferation, and egress [6, 8]. Inclusion membrane proteins (Incs) are a large group of Chlamydiae T3S effectors that share characteristic hydrophobic domains and insert in the chlamydial vacuolar membrane [12, 13]. Other Chlamydiae effectors have been shown to be dispersed in the host cell cytosol [14, 15], or to localize at the nucleus [15, 16], Golgi [17], lipid droplets [18], or plasma membrane [17, 19]. In *C. trachomatis*, about 60 effectors have been identified and many more are expected to exist [8]. Some of these effectors have been shown to be conserved among Chlamydiaceae [14–16, 20, 21], while others have been described to be specific to *C. trachomatis* or to a restricted number of *Chlamydia* species [21]. However, these searches for the presence of homologous effector genes in Chlamydiaceae only considered a few genes, and many were done before the disclosure of the currently known genome sequences.

We previously identified a *C. trachomatis* effector, named CteG, that does not show significant similarity to other proteins except putative homologues within Chlamydiaceae and localizes at the host cell Golgi and plasma membrane at distinct stages of infection [17]. More recently, we showed that CteG promotes host cell lytic exit of *C. trachomatis* by a yet unknown mechanism [22]. Furthermore, CteG binds the host cell centrosomal protein centrin-2 and promotes centrosomal duplication [23]. In a previous work, we identified putative CteG homologs among Chlamydiaceae by performing preliminary Position-Specific Iterated (PSI)-protein BLAST (PSI-BLAST) analyses [17]. In this work, we show that *cteG* gene has over 60 homologs in Chlamydiaceae, including several inparalogs and outparalogs, indicating a complex evolution unique among *C. trachomatis* effectors that is marked by several gene duplication and gene loss events. Although the vast majority of the CteG homologs show modest amino acid sequence similarity to *C. trachomatis* CteG, many of them are also T3S substrates, and can be delivered by *C. trachomatis* into host cells where they also localize at the Golgi region and cell periphery.

## Materials and methods

### Genome data

Publicly available assembled genomes and respective annotations for each *Chlamydia* or *Chlamydiifrater* species analysed (as of June 2023) were retrieved from the National Center for Biotechnology Information (NCBI).

The reference strains used for the analyses and their respective GenBank genome assembly accession numbers are the following: *C. trachomatis* serovar L2 strain 434/Bu (L2/434; GCA_000068585.1); *C. abortus* strain S26/3 (GCA_000026025.1); *C. avium* strain 10DC88 (GCA_000583875.1); *C. buteonis* str. IDL17-4553 (GCA_019056495.1); *C. caviae* strain GPIC (GCA_000007605.1); Candidatus *C. corallus* (referred as *C. corallus*) strain G3/2742 − 324 (GCA_002817655.1); *C. crocodili* str. 12 (GCA_018343815.1); *C. felis* strain Fe/C-56 (GCA_000009945.1); *C. gallinacea* strain 08-1274/3 (GCA_000471025.2); *Candidatus* C. ibidis [referred as *C. ibidis*] strain 10-1398 (GCA_000454725.1); *C. muridarum* strain Nigg (GCA_000006685.1); *C. pecorum* strain E58 (GCA_000204135.1); *C. poikilothermis* strain S15-834 K (GCA_900239975.1); *C. pneumoniae* strain CWL029 (GCA_000008745.1); *C. psittaci* strain 6BC (GCA_000204255.1); *Candidatus* C. sanzinia [referred as *C. sanzinia*] strain 2742 − 308 (GCA_001653975.1); *C. serpentis* strain H15-1957–10 C (GCA_900239945.1); *C. suis* strain MD56 (GCA_000493885.1); *C. abortus* strain 15-70d24 (GCA_900416725.2); *Chlamydiifrater volucris* sp. nov. (referred as *C. volucris*) strain 15-2067_O50 (GCA_902806995.1); *Chlamydiifrater phoenicopteri* sp. nov. (referred as *C. phoenicopteri*) strain 14-2711_R47 (GCA_902807005.1). Species of the Chlamydiaceae sister clade Chlamydiae Clade IV (CC-IV) [24] were also included: Chlamydiae bacterium isolate K940_chlam_9 (GCA_011064985.1), Chlamydiae bacterium isolate KR126_chlam_2 (GCA_011064935.1), Chlamydiae bacterium isolate K1000_chlam_4 (GCA_011065205.1). Closest related species *Candidatus* Protochlamydia naegleriophila strain KNic (GCA_001499655.1), *Estrella lausannensis* strain CRIB 30 (GCA_900000175.1) and *Simkania negevensis* strain Z (GCA_000237205.1) were used as outgroups.

### Reciprocal tBLASTx screen

Putative *cteG* homologs were preliminarily searched by tBLASTx searches using the nucleotide sequence of *cteG* (*ctl0360*) from *C. trachomatis* L2/434 as query against the genome databases created for each chlamydial species. Genomic regions of each species encoding proteins showing similarities with proteins encoded by *cteG* were considered potential hits when e-values < 0.01. Then, the nucleotide sequence corresponding to 2 kbp upstream from the start and 2 kbp downstream from the end of these genomic regions was used for ab initio gene prediction with AUGUSTUS [25], using *Staphylococcus aureus* as the bacterial reference organism. The amino acid sequence of proteins potentially encoded by genes predicted at these regions were used in protein BLASTp searches against the NCBI non-redundant standard database of the corresponding *Chlamydia* or *Chlamydiifrater* species. Whenever the best hit protein in NCBI corresponded to the identity of the protein encoded by the query gene, it was assumed that the gene was present.

To verify putative homology, the nucleotide sequence of the putative *cteG* homolog genes found in the first search was used in a reciprocal tBLASTx against the genome sequence of *C. trachomatis* L2/434. If *cteG* appeared as the top hit with an e-value < 0.01, then the gene was considered a putative *cteG* homolog. A similar procedure was used to identify putative homologs of genes encoding other *C. trachomatis* effectors.

### Phylogenetic analyses

To reconstruct the species tree, single copy orthologs (SCO) were retrieved using Orthofinder 2 [26] (-M msa -S blast -A mafft) from the predicted proteomes of the studied species. The resulting concatenated alignment produced by Orthofinder contained 198,493 amino acid positions that were subsequently used to infer a ML tree with IQ-TREE v1.6.11 [27] using an automatic detection of the best-fitting model of amino acid evolution and an ultrafast bootstrap method (-bb 1,000) [28]. Five independent tree searches were performed in total (--runs 5) and the tree with the highest likelihood score was considered the one representing the most likely phylogenetic relationships between species.

For the CteG phylogeny, putative CteG homologs were obtained through BLASTp searches against the local proteome databases using the CteG amino acid sequence from *C. trachomatis* L2/434 as query (AGJ64459.1; identical to CAP03800.1). A preliminary phylogenetic tree was constructed in IQ-TREE v. 1.6.11 [27] using all top BLASTp hits (e-value < 0.01). As putative CteG sequences were identified in *Chlamydia* species distantly related to *C. trachomatis*, the amino acid sequence of a putative CteG homolog from *C. caviae* (AAP05046.1) was subsequently used in a second BLASTp search. All top BLASTp hits obtained using both blast searches were analysed (e-value < 0.1), except for one sequence from *Chlamydiifrater volucris* (WP_213319067.1) which did not return *cteG* as top hit by reciprocal blast. Redundant sequences were removed with CD-HIT v4.6.7 [29]. The resulting sequences were aligned with MAFFT v. 7.407 [30] using the iterative refinement method L-INS-i (--localpair). Poorly aligned regions were removed with trimAl v1.2 using the “gappyout” option [31]. Phylogenies were constructed for both trimmed and not trimmed alignments. The Maximum Likelihood (ML) trees were inferred with IQ-TREE v1.6.11 [27] using an automatic detection of the best-fitting model of amino acid evolution and ultrafast bootstraps (-bb 1,000) [28]. A total of five runs (-runs 5) were conducted and the tree with the highest likelihood was selected.

### Synteny analyses

Synteny conservation across *Chlamydia* and *Chlamydiifrater* species was done by first identifying genes in the neighbourhood of c*teG* homologs within Chlamydiaceae. This was done either manually using the available Genbank (NCBI) genome assemblies or by ab initio prediction using AUGUSTUS [25]. For each protein potentially encoded by the predicted genes, its identity to a putative *C. trachomatis* homolog was verified by top hit BLASTp and e-value < 0.001.

### Escherichia coli and *Yersinia enterocolitica* strains and growth conditions

*Escherichia coli* NEB^®^ 10β (New England Biolabs) was used for plasmid construction and purification, and *E. coli* ER2925 (New England Biolabs) was used to replicate and purify plasmids for transformation of *C. trachomatis*. *Yersinia enterocolitica* ΔHOPEMT (MRS40 pIML421 [*yopH*_Δ1− 352_, *yopO*_Δ65− 558_, *yopP*_23_, *yopE*_21_, *yopM*_23_, *yopT*_135_]), deficient for the *Yersinia* Yop T3S effectors H, O, P, E, M, and T, but T3S-proficient [32] and T3S-deficient *Y. enterocolitica* ΔHOPEMT ΔYscU (MRS40 pFA1001 *yopH*_Δ1− 352_, *yopO*_Δ65− 558_, *yopP*_23_, *yopE*_21_, *yopM*_23_, *yopT*_135_], *yscU*_Δ1− 354_) [33] were used for T3S assays. The *yscU* gene encodes an essential component of the *Y. enterocolitica* T3S system, and the *yscU*_Δ1− 354_ mutation is non-polar [34]. *E. coli* and *Y. enterocolitica* strains were grown in liquid or agar lysogeny broth (LB; NZYTech) with the appropriate selective antibiotics and supplements. *E. coli* and *Y. enterocolitica* cells were transformed with the plasmids by electroporation. T3S assays using *Y. enterocolitica* were done as previously described [34, 35].

### Plasmids, DNA oligonucleotides, and DNA manipulations

The plasmids used in this work are listed in Table S1. The DNA oligonucleotides used in plasmid construction and in other molecular biology procedures are listed in Table S2. In general, plasmids were generated by cloning with restriction enzymes using standard molecular biology procedures. Briefly, DNA sequences were amplified with proof-reading Phusion high-fidelity DNA polymerase (Thermo Fisher Scientific). DNA sequences and backbone plasmids were then digested with FastDigest restriction enzymes (Thermo Fisher Scientific) and ligated with T4 DNA ligase (Thermo Fisher Scientific). NZYTaq II DNA polymerase (NZYTech) was used for screening of positive clones. DNA fragments and plasmids were purified with DNA Clean & Concentrator-5TM kit (Zymo Research), ZymocleanTM Gel DNA recovery kit (Zymo Research), NZYMiniprep kit (NZYTech) or NZYMidiprep kit (NZYTech) following the manufacturer’s instructions. In general, pLJM3 [36] was used as vector to generate plasmids for protein production and analysis of T3S in *Y. enterocolitica*; plasmids pSVP246 [17] and pMC114 (generated in this work), both derivatives of p2TK2-SW2 [37], were used as vectors to generate plasmids for protein production and analysis of delivery into host cells by *C. trachomatis*. To generate plasmids encoding *cteG* homologs we used genomic DNA, kindly provided by Agathe Subtil (*C. caviae* and *C. pneumoniae*), Ian Clarke (*C. muridarum*), María Rosa Vergara (*C. abortus*), and Nicole Borel (*C. pecorum* and *C. suis*), as template for the PCR reactions. The accuracy of the nucleotide sequence of all the inserts in the constructed plasmids was checked by DNA sequencing (done at STAB VIDA).

### Mammalian cell lines

HeLa 229 cells (from the European Collection of Authenticated Cell Culture; ECACC) were passaged in 4.5 g/L glucose, L-glutamine Dulbecco’s Modified Eagle’s Medium (DMEM; Corning) supplemented with heat-inactivated 10% (v/v) fetal bovine serum (FBS; Thermo Fisher Scientific) at 37ºC in a humidified atmosphere of 5% (v/v) CO_2_ and detached from culture plates or flasks with TrypLE™ Express (Thermo Fisher Scientific). Cell cultures were regularly tested for *Mycoplasma* by conventional PCR as described [38].

### *C. trachomatis* strains and growth conditions

The *C. trachomatis* strains used and generated in this work are listed in Table S3. They were propagated in HeLa 229 cells using standard procedures [39], and as described in our previous studies [17, 22]. *C. trachomatis* transformants were generated essentially as described by Agaisse and Derré (66), and as described in our previous studies [17, 40], except that newly generated *C. trachomatis* strains used for analysis of protein delivery into HeLa 229 cells were not plaque purified. *Chlamydia* stocks were tested for *Mycoplasma* by conventional PCR [38] and DNA sequencing techniques.

### Infection of HeLa cells with *C. trachomatis*

Infection of HeLa cells with *C. trachomatis* for subsequent immunofluorescence and immunoblotting analyses were done as described previously [17, 22]. In experiments where HeLa 229 cells were infected with *C. trachomatis* strains carrying plasmids encoding genes under the control of the *tetA* tetracycline-inducible promoter (Table S3), anhydrotetracycline was added to 50 ng/ml at time zero of infection to process cells at 24 h post-infection (p.i), or at time zero and at 24 h p.i. to process cells at 46 h p.i.

### Immunoblotting and immunofluorescence microscopy

Immunoblotting and immunofluorescence microscopy were performed as previously described [17, 22]. For immunoblotting the following antibodies were used: rat anti-HA (3F10; Roche; 1:1,000), mouse anti-chlamydial Hsp60 (A57-B9; Thermo Fisher Scientific; 1:1,000), mouse anti-α-tubulin (clone B-5-1-2; Sigma-Aldrich; 1:1,000), followed by anti-rat or anti-mouse horseradish peroxidase (HRP)-conjugated secondary antibodies (GE Healthcare and Jackson ImmunoResearch; 1:10,000). For immunofluorescence microscopy the following antibodies and dyes were used: rat anti-HA (3F10; Roche; 1:200), rabbit anti-GM130 (Sigma Aldrich; 1:200), rabbit anti-Cap1 (kindly provided by Agathe Subtil; 1:200) [41], goat anti-*Chlamydia* major outer membrane protein (MOMP) (Abcam; 1:200), and goat anti-*C. trachomatis* FITC-conjugated antibody (Sigma-Aldrich, 1:150), followed by fluorophore-conjugated Rhodamine Red-X-conjugated anti-rat, AF488-conjugated anti-rabbit, DyLight 405-conjugated anti-goat antibodies (Jackson ImmunoResearch; 1:200), and staining with 4′,6-Diamidino-2-phenylindole (DAPI; 1:30,000).

## Results

### Preliminary screening of putative homologs of CteG and of other *C. trachomatis* effectors within Chlamydiaceae

**Fig. 1:**
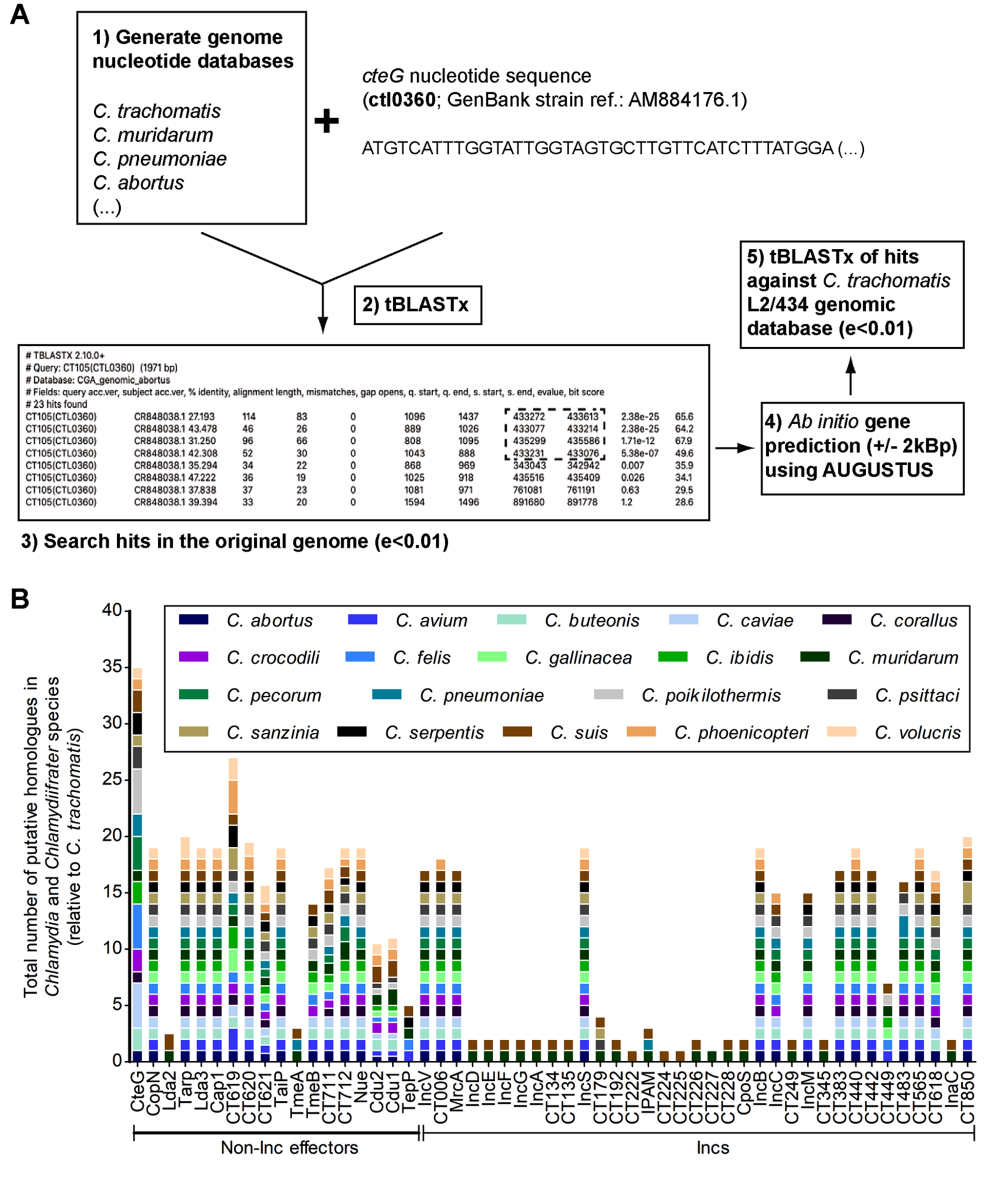
CteG is the *C. trachomatis *effector with the higher relative number of putative homologs within Chlamydiaceae. **(A)** Graphical summary of the reciprocal tBLASTx approach to identify putative homologs of CteG within Chlamydiaceae. A similar procedure was used to identify putative homologs of non-Inc effectors and Incs. **(B)** The number of putative homologs found for each protein and in each *Chlamydia* or *Chlamydiifrater* species is coloured as indicated in the legend above the graph and depicted relative to the number of paralogs in *C. trachomatis*. The length of the coloured rectangles is proportional to the number of putative homologs in each species (e.g., only one homolog in CT222). See Table S4 for details

To reanalyse the distribution of putative CteG homologs across Chlamydiaceae, we started by performing a preliminary tBLASTx analysis against a local genome database. The top hits were subsequently used in a reciprocal tBLASTx against *C. trachomatis*, aiming to further verify putative homology to *cteG*, as illustrated in Fig. 1A. This revealed the presence of 36 putative homologs of CteG (including *C. trachomatis* CteG) encoded within Chlamydiaceae genomes with some species presenting several copies (four in *C. caviae*, *C. felis*, and *C. poikilothermis*; three in *C. pecorum*; and two in *C. buteonis*, *C. crocodili*, *C. ibidis*, *C. pneumoniae*, *C. psittaci*, *C. serpentis*, and *C. suis*) and only two species (*C. avium* and *C. gallinacea*) showing no putative CteG homologs (Table S4). Using this strategy, putative homologs of CteG were not found in species from a sister lineage of the Chlamydiaceae (CC-IV; [24]) or in closely related species belonging to the Chlamydiales/Parachlamydiales orders (*E. lausannensis*, *S. negevensis*, *Ca.* P. naegleriophila), suggesting that the ancestral *cteG* gene appeared after the divergence of Chlamydiaceae from other Chlamydiae families and from sister lineage CC-IV.

As previously described [17], amino acid similarity along the full-length of *C. trachomatis* CteG and its putative homologs was only observed in *C. muridarum* and *C. suis* (Fig. S1). All the other putative homologs of CteG only show sequence similarity to specific regions of CteG, ranging from amino acid residue 143 to the C-termini of CteG (Fig. S1). Furthermore, although *C. buteonis* HBN95_03995, *C. caviae* CCA_00297 and CCA_00298, *C. crocodili* H9Q19_03970, *C. felis* CF_0705 and CF_0706, *C. poikilothermis* C834K_0321 and C834K_0322, *C. serpentis* C10C_1043 were identified in the reciprocal BLAST analysis as putative homologs of CteG, they show a limited range of amino acid similarity (20 to 151 residues) and mostly display low sequence similarity (20–27% of identity; e-values from 0.013 to 3e^− 6^) to CteG (Fig. S1).

A procedure similar to what is illustrated in Fig. 1A was used to search for putative homologs within Chlamydiaceae of 54 additional *C. trachomatis* effectors, including 35 Incs experimentally shown to localize at the inclusion membrane (reviewed in [8] and references therein). Among the chlamydial effectors, most of the DUF582 proteins (CT619, CT620, CT621, CT711, CT712) [15] and the deubiquitinases Cdu1 and Cdu2 [42] led to the detection of different numbers of putative paralogs within *C. trachomatis* (Table S4). Therefore, for a visualization of how putative homologs of CteG and other *C. trachomatis* effectors are spread across Chlamydiaceae, the total number of putative homologs found in each case were plotted relative to the number of corresponding putative paralogs identified in *C. trachomatis* (Fig. 1B). Among the *C. trachomatis* effectors analysed, CteG displayed the higher relative number of putative homologs within Chlamydiaceae (Fig. 1B). By comparison to *C. trachomatis*, this is because of the existence of multiple CteG putative homologs in several Chlamydiaceae species, which is not seen to such an extent for any other *C. trachomatis* effector (Fig. 1B). This was intriguing and prompted us to analyse in further detail the evolutionary history of CteG, and if some of the putative CteG homologs are also T3S substrates delivered into host cells during infection.

### Evolution of CteG in Chlamydiaceae

**Fig. 2:**
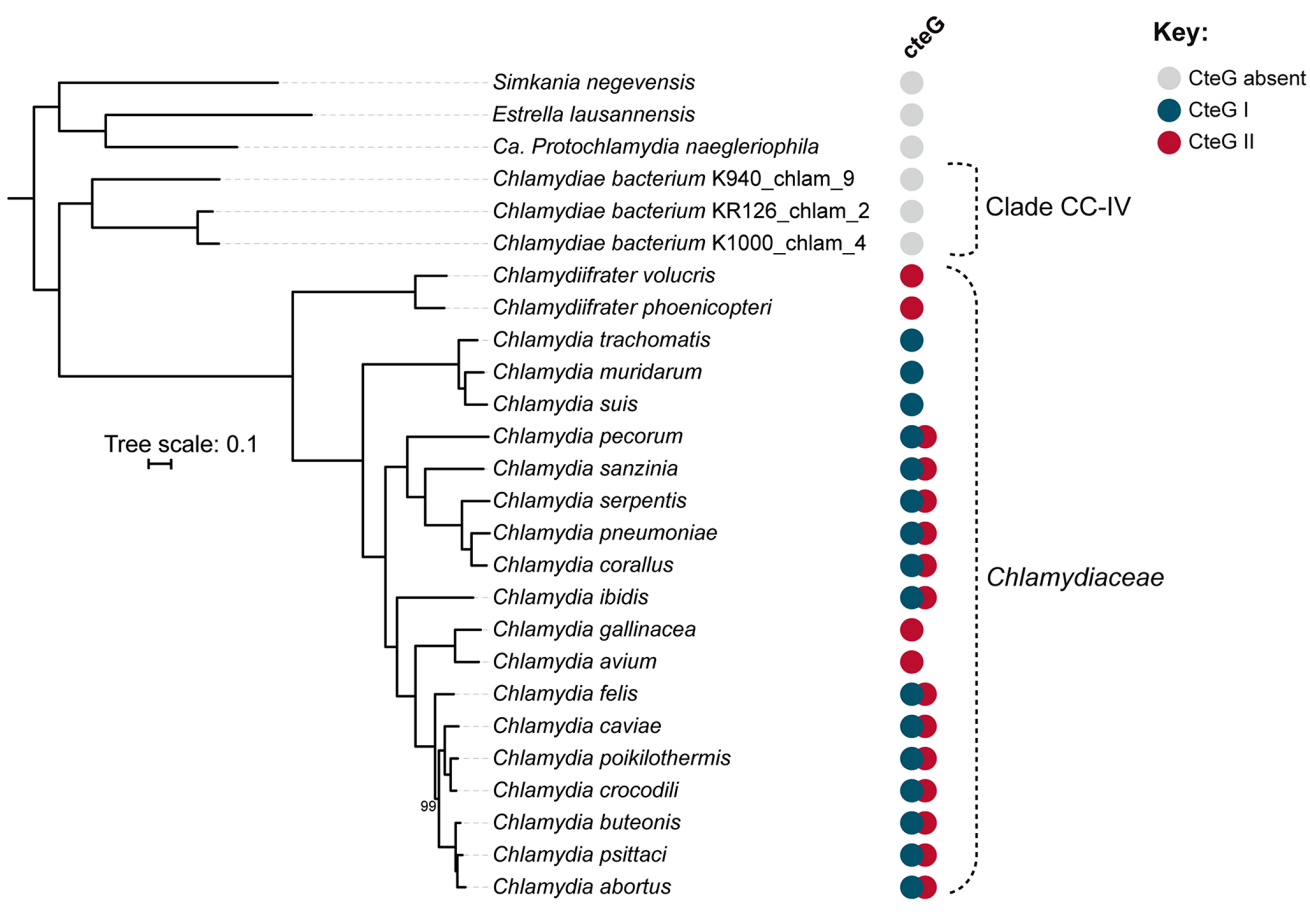
Phylogenomic tree of the Chlamydiaceae and closest related species of the Chlamydiae Clade IV (CC-IV) clade. The phylogeny was constructed with 214 single copy orthologs and rooted with *S. negevensis*, *E. lausannensis* and *Ca*. P. naegleriophila. Only bootstrap values below 100 are shown next to the respective nodes. Presence and absence of CteG homologs is illustrated as indicated next to each species name according to the local BLASTp analyses using the respective whole proteomes (see Fig. 3). Blue represents CteG belonging to clade I, red CteG belonging to clade II, and white means that CteG was absent, as also shown in Fig. 3. Original tree files and alignments can be found in Figshare: https://figshare.com/s/b5ee1bbacc3d9ec347fb

To deepen the study of the evolution of *cteG* among Chlamydiaceae species and closest relatives, and recover the highest number of homologs possible, we next inferred a species tree of the Chlamydiaceae (Fig. 2) and searched for all putative CteG homologs in a local proteome database, lowering the stringency of the BLASTp search (e-value < 0.1). The species tree was constructed using a phylogenomic approach, consisting of an alignment of 214 concatenated proteins obtained from Orthofinder (Fig. 2). The phylogenetic relationships among the Chlamydiaceae members is either consistent [43] or slightly differs from that described in previous studies [3, 24, 44]. Such variations could be attributed to the inclusion of more chlamydial species or to the use of different strategies for phylogenetic inference.

**Fig. 3:**
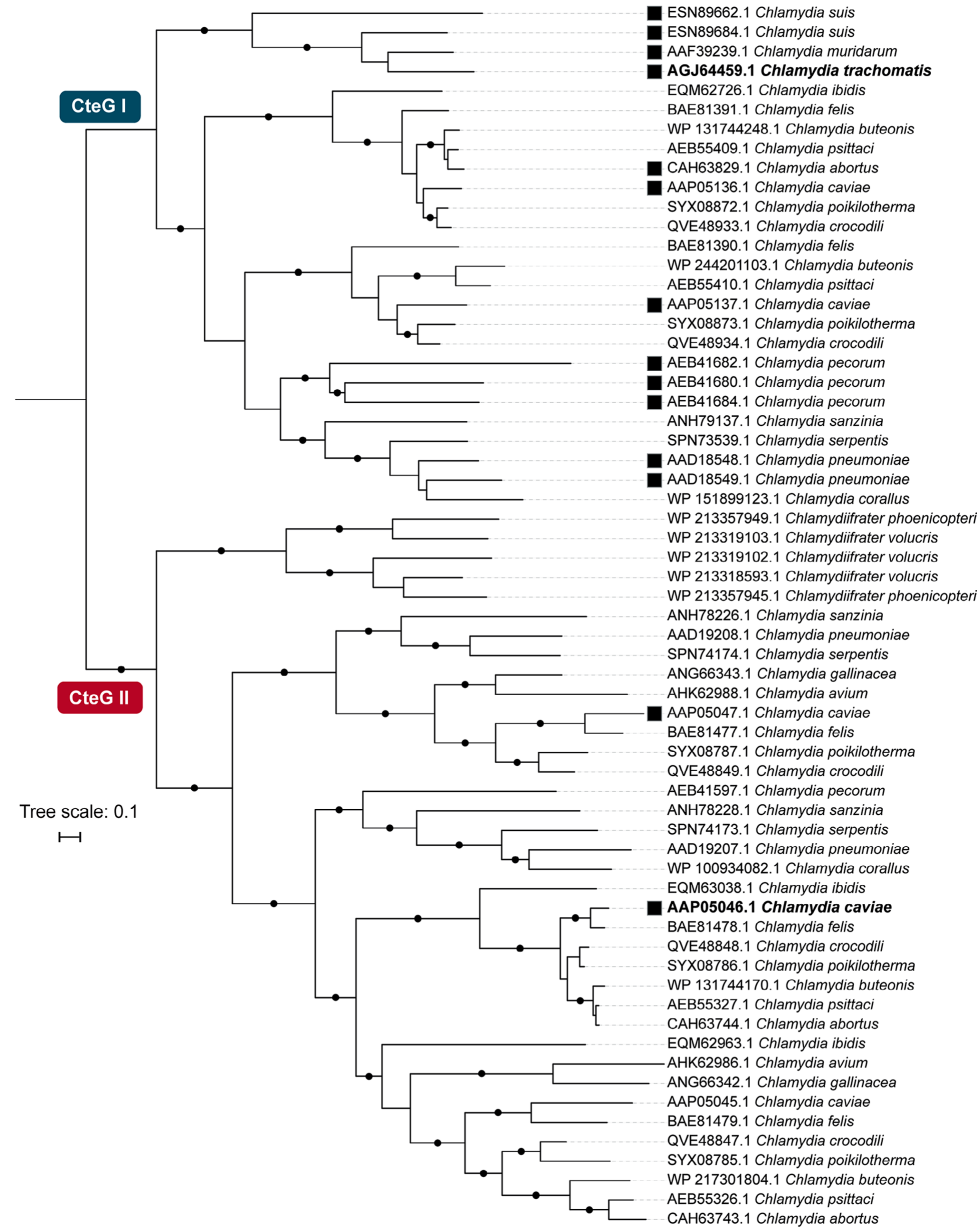
Evolutionary history of CteG across Chlamydiaceae and closest relatives. CteG phylogeny comprising all top CteG BLASTp hits (e-value < 0.1). The two sequences used as queries for the BLASTp searches are highlighted in bold. Proteins selected for further analyses of protein secretion and delivery into host cells are highlighted by black filled squares. Phylogeny was midpoint rooted using iTOL v5 [71]. Only bootstrap values higher than 95 are shown as black dots next to the respective nodes. Phylogenies were visualized and rooted using iTOL [71]. Original tree files and alignments can be found in Figshare: https://figshare.com/s/b5ee1bbacc3d9ec347fb

We next inferred the phylogenetic tree of CteG using all putative homologs recovered by the local BLASTp search (total of 63 sequences, including *C. trachomatis* CteG; Fig. 3 and Table S5). The phylogeny separated the CteG homologs in two main clades: CteG I (*C. trachomatis* CteG and 25 other proteins) and CteG II (37 proteins). Clade CteG I includes CteG from *C. trachomatis* and putative orthologs from closely related species, grouping essentially according to the species phylogeny (Figs. 2 and 3). However, and in line with our initial screen for CteG homologs (Fig. 1), several paralogs could be identified. These include inparalogs (e.g., in *C. pecorum*) and outparalogs (e.g., in *C. cavie*, *C. crocodili* or *C. poikilothermis*). This suggests that several *cteG* duplication events occurred at different time points during the evolution of Chlamydiaceae. Clade CteG II includes more distantly related homologs from Chlamydiaceae species, but not from *C. trachomatis* and closest relatives *C. suis* and *C. muridarum*. Interestingly, this clade also includes CteG homologs from distantly related *Chlamydiifrater* species, which are seemingly absent from CteG I clade. As in CteG I, in CteG II, the global relationships between species/clades follow those from the species tree (Figs. 2 and 3). However, inparalogs (*C. volucris*) and several outparalogs (e.g., in *C. abortus*, *C. caviae*, or *C. poikilothermis*) could also be inferred (Fig. 3).

There are differences in the number and identity of putative CteG homologs retrieved from the preliminary reciprocal tBLASTx approach (Fig. 1A and Table S4) and the homologs identified by the BLASTp and subsequent CteG phylogeny (Fig. 3 and Table S5). However, the expansion of *cteG*-related genes among Chlamydiaceae suggested by the initial screen revealed to be more significative with the more robust analysis of CteG phylogeny: 63 CteG homologs (including *C. trachomatis* CteG) instead of the 35 putative homologs initially found (Figs. 1 and 3). Only *C. trachomatis* and *C. muridarum* do not possess CteG paralogs encoded in their genomes. All other Chlamydiaceae species encode 2 (*C. suis*, *C. corallus*, *C. avium*, *C. gallinacea*, *C. phoenicopteri*), 3 (*C. serpentis*, *C. sanzinia*, *C. abortus*, *C. ibidis*, *C. volucris*), 4 (*C. pneumoniae*, *C. pecorum*, *C. buteonis*, *C. psittaci*), or 5 (*C. crocodili*, *C. poikilotermis*, *C. caviae*, *C. felis*) proteins evolutionarily related to *C. trachomatis* CteG. As *cteG* homologs show significant divergence among each other it is plausible that other putative *cteG* homologs were overlooked using our approach. However, we constructed an HMM profile for CteG using the 62 homologs (recovered by BLASTp), used a phmmer-based search [45] and did not find additional homologs. Overall, when considering the distribution (Fig. 2) and phylogenetic relationships (Fig. 3) of CteG proteins, it seems plausible that an ancient duplication in the most recent common ancestor (MRCA) of the Chlamydiaceae took place originating two distinct *cteG* ‘alleles’, followed by multiple gene duplication and differential loss events.

### Synteny of *cteG* homologs

**Fig. 4:**
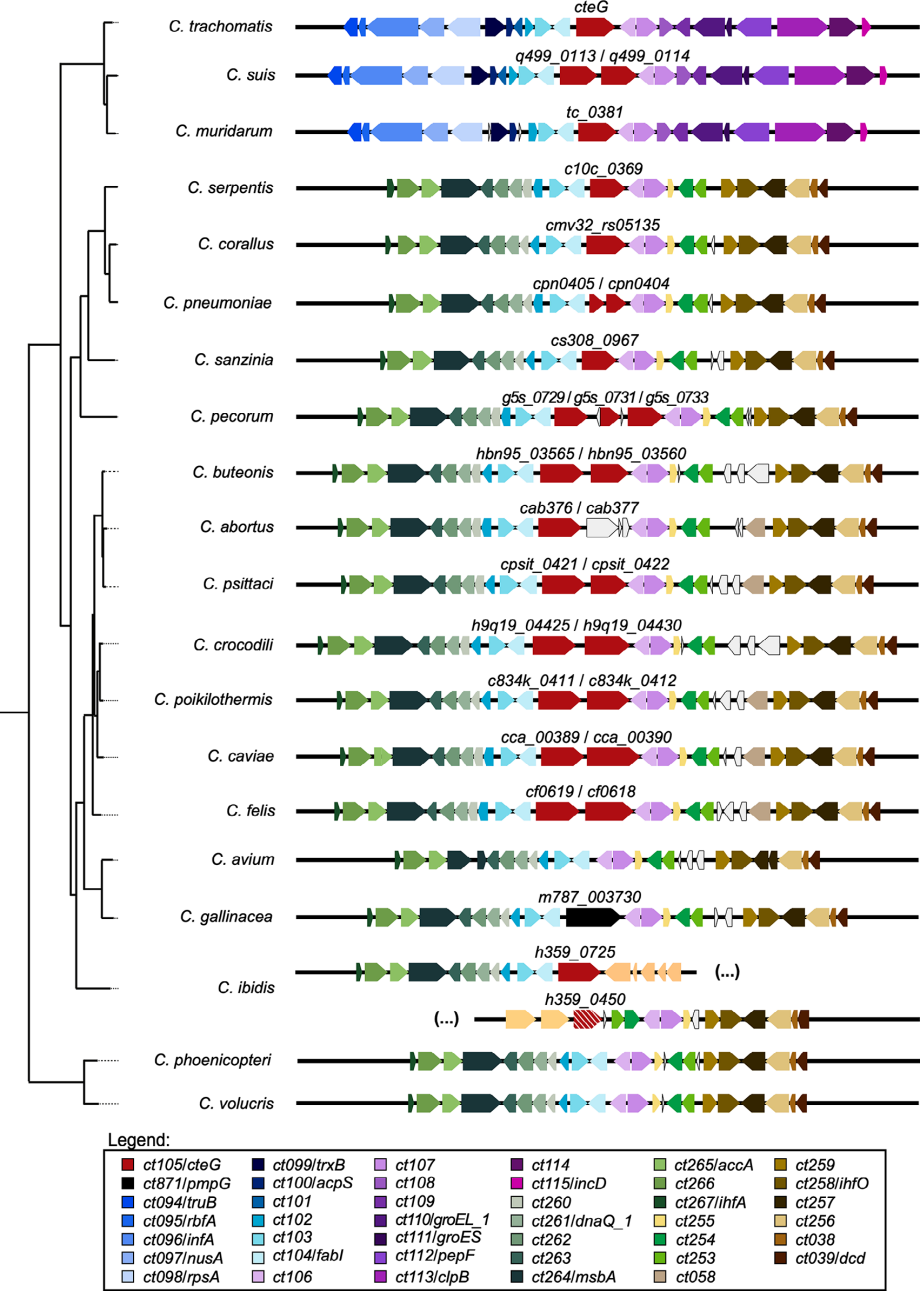
Genomic region of *cteG* and of each of its syntenic homologs in other *Chlamydia* and *Chlamydiifrater* species. *cteG* homologs identified by the analysis of CteG phylogeny depicted in Fig. 2 are coloured in red. Our preliminary search for putative CteG homologs (Fig. 1 and Table S4) retrieved a *C. ibidis* protein (H359_0450) that was not retrieved by the CteG phylogeny. The corresponding gene is indicated coloured in red and white stripes. Other genes are coloured as indicated within the figure. Genes for which no putative homologs were found are coloured in white. *C. abortus cab377* was not identified as a putative homolog of *cteG* and is annotated in NCBI databases as a fragmented pseudogene. Genomic regions are depicted according to the species tree in Fig. 2A. The nomenclature in the legend for each group of putative homologs is from *C. trachomatis* D/UW3 strain [11]

We next analysed gene organization in the vicinity of the *cteG* homologs. While the analysis was performed based on the genomic sequence of the *C. trachomatis* L2/434 strain, we used the nomenclature of the *C. trachomatis* serovar D UW3 strain (D/UW3). The genomic nucleotide sequences of the two strains are syntenic and 99.55% identical [46], and as strain D/UW3 corresponds to the first sequenced chlamydial genome [11] its gene nomenclature is often used as reference. The analysis revealed the existence of homologs that are syntenic to *cteG* (*ct105* in *C. trachomatis* D/UW3) and that almost all localize in a locus encompassing *C. trachomatis ct102* to *ct107* homologous genes (Fig. 4). They all correspond to the CteG I clade in the phylogenetic tree of CteG, suggesting they are all *cteG* orthologs (Fig. 3). In addition, the genes encoding the CteG homologs corresponding to the CteG II clade in the phylogenetic tree of CteG (Fig. 3) are non-syntenic to *C. trachomatis cteG* (Fig. S2). However, they are all syntenic with each other occupying a locus flanked by homologs of *C. trachomatis ct009-ct008-ct007* on one side (except *C. corallus*, *C. pneumoniae*, and *C. serpentis* where only homologs of *ct009-ct008* were found), and tRNA-Ser and *ct356*-*ct355-ct354* (most *Chlamydia* species) or tRNA-Ser and *ct003-ct002*-*ct001* (*C. serpentis*, *C. corallus*, and *C. pneumoniae*) on the other side (Fig. S2). In the *Chlamydiifrater* species, the locus containing the CteG homologs also includes several *pmp* (polymorphic membrane protein) genes [47] and homologs of *ct009*,* ct008*, and *ct007* with altered localization relative to *C. trachomatis* (Fig. S2). This locus is flanked by homologs of *ct356*-*ct355-ct354* on one side and of *ct004*-*ct003-ct002*-*ct001* on the other side (Fig. S2), thus showing further rearrangements relative to the syntenic locus in most *Chlamydia* species.

In summary, the synteny of *cteG* homologs (from the CteG I and CteG II clades) within Chlamydiaceae further supports that they are evolutionarily related.

### Most CteG homologs within Chlamydiaceae are T3S substrates

To analyse if the CteG homologs identified within Chlamydiaceae are also T3S substrates, we selected both CteG I and CteG II proteins and tested if they can be secreted as full-length proteins by *Yersinia enterocolitica*. Bacteria such as *Shigella flexneri* or *Yersinia* spp. with well-characterized T3S systems were used as heterologous systems to show that *Chlamydia* encodes T3S substrates [10, 12], and have been used to screen for putative chlamydial effectors [35, 48, 49] and to test if specific chlamydial proteins are T3S substrates [41, 50–53].

The CteG homologs analysed were from *C. muridarum* (AAF39239.1/TC_0381), *C. suis* (ESN89684.1/Q499_0113, ESN89662.1/Q499_0114), *C. pneumoniae* (AAD18548.1/Cpn_0404, AAD18549.1/Cpn_0405), *C. abortus* (CAH63829.1/CAB376), *C. pecorum* (AEB41680.1/G5S_0729, AEB41682.1/G5S_0731, AEB41684.1/G5S_0733) and *C. caviae* (AAP05046.1/CCA_00297, AAP05047.1/CCA_00298, AAP05136.1/CCA_00389, AAP05137.1/CCA_00390). This includes CteG I proteins (TC_0381, Q499_0113, which are closely related to CteG; and Q499_0114, Cpn_0404, Cpn0405, CAB376, G5S_0729, G5S_0731, G5S_0733, CCA_00389, CCA_00390, which by comparison to TC_0381 and Q499_0113 show less similarity to CteG) and CteG II proteins (CCA_00297, CCA_00298) (Fig. 3 and Fig. S1).

We first generated plasmids enabling gene expression from the *Yersinia yopE* T3S effector gene promoter of CteG homologs with a C-terminal haemagglutinin (HA) epitope tag. While generating the plasmids encoding *C. suis* Q499_0114-HA and *C. pecorum* G5S_0729-HA, we noted by DNA sequencing that the genes were truncated relative to what is annotated in the NCBI database. For *C. suis* Q499_0114 we generated two plasmids encoding two putative proteins that we named Q499_0114A (residues 1 to 283 of the annotated Q499_0114 sequence) and Q499_0114B (residues 314 to 529 of the annotated Q499_0114 sequence) while for *C. pecorum* G5S_0729 we only analysed the truncated protein (residues 1 to 120 and 274 to 337 of the annotated sequence).

**Fig. 5:**
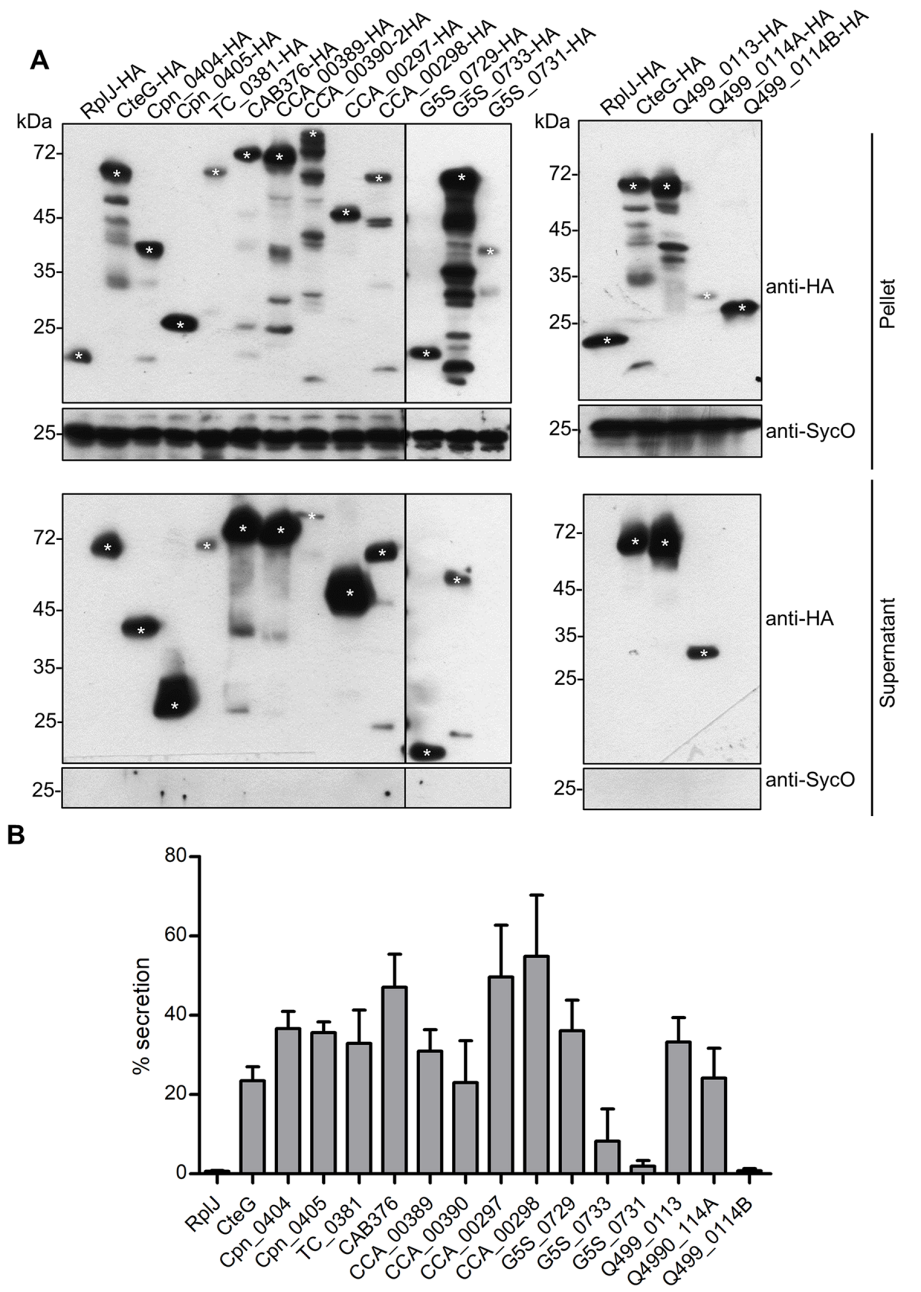
Analysis of Type III secretion (T3S) of CteG homologs within Chlamydiaceae using *Y. enterocolitica* as heterologous host. **(A)** Type III secretion (T3S)-proficient *Y. enterocolitica* ΔHOPEMT was used to analyse secretion of CteG homologs within Chlamydiaceae with a C-terminal HA epitope tag. Immunoblots show the result of T3S assays in which proteins in culture supernatants (S, secreted proteins) and in bacterial pellets (P, non-secreted proteins) from ∼ 5 × 10^8^ and ∼ 5 × 10^7^ bacteria, respectively, were loaded per lane. CteG, a known *C. trachomatis* T3S substrate [35], was used as positive control, and the *C. trachomatis* ribosomal protein RplJ was used as a negative control [35]. The bands corresponding to the predicted molecular mass of the proteins analysed are indicated with a white asterisk (RplJ-HA, ∼ 19 kDa; CteG-HA, ∼ 68 kDa; Cpn_0404-HA, ∼ 37 kDa; Cpn_0405-HA, ∼ 26 kDa; TC_0381-HA, ∼ 71 kDa; CAB376-HA, ∼ 80 kDa; CCA_00389-HA, ∼ 79 kDa; CCA_00390-HA, ∼ 94 kDa; CCA_00297-HA, ∼ 49 kDa; CCA_00298-HA, ∼ 60 kDa; G5S_0729-HA, ∼ 19 kDa; G5S_0733-HA, ∼ 63 kDa; G5S_0731-HA, ∼ 38 kDa; Q499_0113-HA, ∼ 68 kDa; Q499_0114A-HA, ∼ 29 kDa; Q499_0114A-HA, ∼ 32 kDa). SycO is a strictly cytosolic *Yersinia* T3S chaperone [72] and its immunodetection ensured that the presence of HA-tagged proteins in the culture supernatants was not a result of bacterial lysis or contamination. **(B)** The percentage (%) of secretion of each protein by *Y. enterocolitica* ΔHOPEMT was calculated by densitometry of the bands in the immunoblots, as the ratio between the amount of secreted and total protein. Data are the mean ± standard error of the mean from at least 3 independent experiments

The plasmids encoding HA-tagged CteG homologs were then separately introduced into T3S-proficient *Y. enterocolitica* (ΔHOPEMT), which lacks most *Yersinia* effectors [32, 33, 35]. As positive and negative controls in the secretion assays, we used *Y. enterocolitica* strains encoding CteG-HA and a HA-tagged chlamydial ribosomal protein (RplJ-HA), respectively. In general, the proteins migrated according to their predicted molecular mass (Fig. 5A and B). Many CteG homologs revealed multiple bands besides the band corresponding to the predicted molecular mass (Fig. 5A and B), a feature of unknown relevance that we consistently found in our studies involving CteG [17, 22, 35]. This revealed that 11 out of the 14 (∼ 80%) CteG homologs tested were secreted except for *C. suis* Q499_0114B and *C. pecorum* G5S_0731, which were not secreted, and for C. *pecorum* G5S_0733, where the results were unclear (Fig. 5A and B; summarized in Table 1). Then, the plasmids encoding the HA-tagged CteG homologs that were secreted (including the plasmid encoding G5S_0733) were individually introduced into T3S-deficient *Y. enterocolitica* ΔHOPEMT ΔYscU. Assays with the resulting strains confirmed that secretion of these CteG homologs was T3S-dependent (Fig. S3). In summary, these data suggest that, overall, the CteG homologs within Chlamydiaceae are also T3S substrates, even those more distantly related such as *C. caviae* CCA_00297 and CCA_00298.

**Table 1:** Summary of the data on type III secretion and delivery into host cells of CteG homologs within Chlamydiaceae analysed in this study

*Chlamydia* species	Protein (with C_ter_ 2HA)	Type III secreted by Ye^a^?	Delivered by Ctr^b^ into host cells?		Subcellular localization^c^	
			24 h p.i.	46 h p.i.	24 h p.i.	46 h p.i.
*C. trachomatis*	CteG	Yes	Yes	Yes	G	PM
*C. muridarum*	TC_0381	Yes	Yes	Yes	G	G, PM
*C. suis*	Q499_0113	Yes	Yes	Yes	G, PM	PM
	Q499_0114A	Yes	No	No	ND	ND
	Q499_0114B	No	NT^d^	NT	NT	NT
*C. pneumoniae*	Cpn_0404	Yes	No	No	ND	ND
	Cpn_0405	Yes	No	No	ND	ND
*C. abortus*	CAB376	Yes	No	Yes	ND	PM
*C. pecorum*	G5S_0729	Yes	No	No	ND	ND
	G5S_0731	No	NT	NT	NT	NT
	G5S_0733	Yes?	No	Yes	ND	G
*C. caviae*	CCA_00297	Yes	Yes	Yes	G, PM	G, PM
	CCA_00298	Yes	Yes	Yes	G, PM	G, PM
	CCA_00389	Yes	No	Yes	ND	PM
	CCA_00390^e^	Yes	NT	NT	NT	NT

^a^Ye, *Yersinia enterocolitica*; ^b^Ctr, *Chlamydia trachomatis*; ^c^Detected in the host cell Golgi (G), or plasma membrane (PM), or not delivered into host cells (ND); ^d^NT, Not tested;^e^*C. trachomatis* transformants were not obtained with plasmid encoding CCA_00390-2HA

### Many CteG homologs can be delivered into host cells by *C. trachomatis*

Next, we sought to test if the CteG homologs that are type III secreted by *Y. enterocolitica*, including G5S_0733, could be delivered by *C. trachomatis* into infected host cells. To avoid possible toxic effects in *C. trachomatis*, we initially generated plasmids where the expression of the genes encoding the CteG homologs was under control of the tetracycline-inducible *tetA* promoter. In all cases, the constructs were designed for the CteG homologs to have a C-terminal double (2HA) epitope tag. After individual introduction of the plasmids into CteG-deficient *C. trachomatis* (*cteG::aadA*), we successfully obtained transformants in eleven of the twelve cases. The exception was for the strain that should encode CCA_00390-2HA, and this protein was not further analysed.

To confirm that the generated *C. trachomatis* strains were producing the desired proteins, HeLa cells were individually infected for 24 and 46 h with each of the eleven strains, and with a derivative of the *cteG::aadA* mutant harbouring a plasmid encoding CteG-2HA, also expressed from the *tetA* promoter [CteG-2HA(*P*_*tetA*_)]. Immunoblotting of whole cell extracts confirmed the production of Q499_0113-2HA, Cpn0404-2HA, Cpn0405-2HA, CAB376-2HA, G5S0729-2HA, G5S0733-2HA, CCA_00297-2HA, CCA_00298-2HA, and CCA_00389-2HA (Fig. S4). However, we could not detect production of TC_0381-2HA and Q499_0114A-2HA. Alternatively, we generated strains derived from the *cteG::aadA* mutant harbouring plasmids encoding these two proteins expressed from the *cteG* promoter. Immunoblotting from whole cell extracts of HeLa cells infected for 24 and 46 h with these two strains, and with a derivative of the *cteG::aadA* mutant harbouring a plasmid encoding CteG-2HA expressed from its own promoter [CteG-2HA(*P*_*cteG*_)], confirmed the production of both TC_0381-2HA and Q499_0114A-2HA (Fig. S5). As previously observed in Fig. 5A and B and in previous studies of *C. trachomatis* CteG [17, 22, 35], the detection by immunoblotting of the production of many of its homologs in Chlamydiaceae revealed multiple bands besides the band corresponding to the predicted molecular mass (Figs. S4 and S5).

**Fig. 6:**
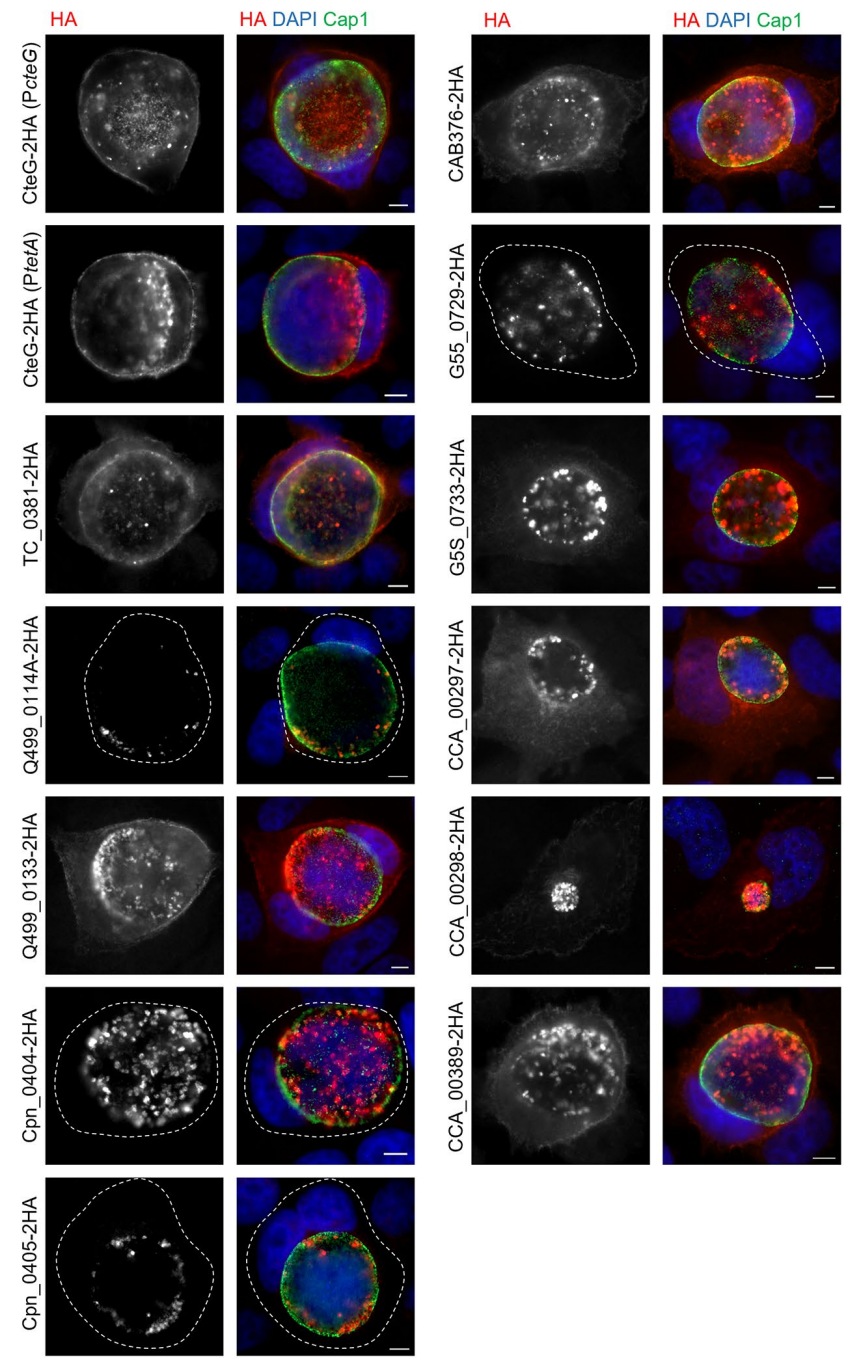
Delivery into host cells by *C. trachomatis* of CteG homologs in Chlamydiaceae. HeLa cells were infected for 46 h with *C. trachomatis cteG::aadA* harboring plasmids encoding CteG or CteG homologs within Chlamydiaceae (Q499_0113 and Q499_0114A, from *C. suis*; Cpn0404 and Cpn0405, from *C. pneumoniae*; CAB376 from *C. abortus*; CCA00389, CCA00297 and CCA00298, from *C. caviae*; G5S_0733 and G5S_0729, from *C. pecorum*; TC_0381, from *C. muridarum*) with a 2HA C-terminal epitope tag. The gene encoding CteG was expressed from its own promoter (P*cteG*) or from the *tetA* promoter (P*tetA*)) and the genes encoding its homologs within Chlamydiaceae were mostly expressed from P*tetA*, except for the genes encoding TC_0381 and Q499_0114A that were expressed from P*cteG*. Infected cells were fixed with 4% (w/v) paraformaldehyde and immunolabelled with antibodies against HA (red) and the inclusion membrane-localized protein Cap1 (green) and appropriate fluorophore-conjugated secondary antibodies. The host and chlamydial were also stained with DAPI (blue). The immunolabeled and stained cells were analysed by fluorescence microscopy. Scale bars, 5 μm

To analyse if the various proteins were delivered by *C. trachomatis* into host cells, HeLa cells were infected for 24 and 46 h with each of the newly generated strains encoding homologs of CteG and also with the strains producing CteG-2HA(*P*_*tetA*_) and CteG-2HA(*P*_*cteG*_). We then immunolabelled the infected cells with antibodies against HA and Cap1 [a *C. trachomatis* effector known to localize at the inclusion membrane; [54]], followed by fluorophore-conjugated secondary antibodies and DAPI (to stain the DNA in the host cell nucleus and within chlamydiae). Analysis by fluorescence microscopy revealed that among the 11 proteins tested, ∼ 65%/7 (*C. muridarum* TC_0381, *C. suis* Q499_0113, *C. abortus* CAB376, *C. pecorum* G5S_0733, and *C. caviae* CCA_00297, CCA_00298, and CCA_00389) were delivered by *C. trachomatis* into host cells, while for ∼ 35%/4 (*C. suis* Q499_0114A, *C. pneumoniae* Cpn_0404 and Cpn_0405, and *C. pecorum* G5S_0729) their delivery into host cells by *C. trachomatis* was not observed (Fig. 6 and Fig. S6; summarized in Table 1). In a few cases (*C. abortus* CAB376, *C. pecorum* G5S_0733, and *C. caviae* CCA_00389), the proteins were only found to be delivered into host cells at 46 h p.i., but not at 24 h p.i. (Fig. 6 and Fig. S6; summarized in Table 1). The *C. trachomatis* strain producing *C. caviae* CCA_00298 showed a clear growth defect and at 46 h p.i. almost only small inclusions were observed (Fig. 6).

**Fig. 7:**
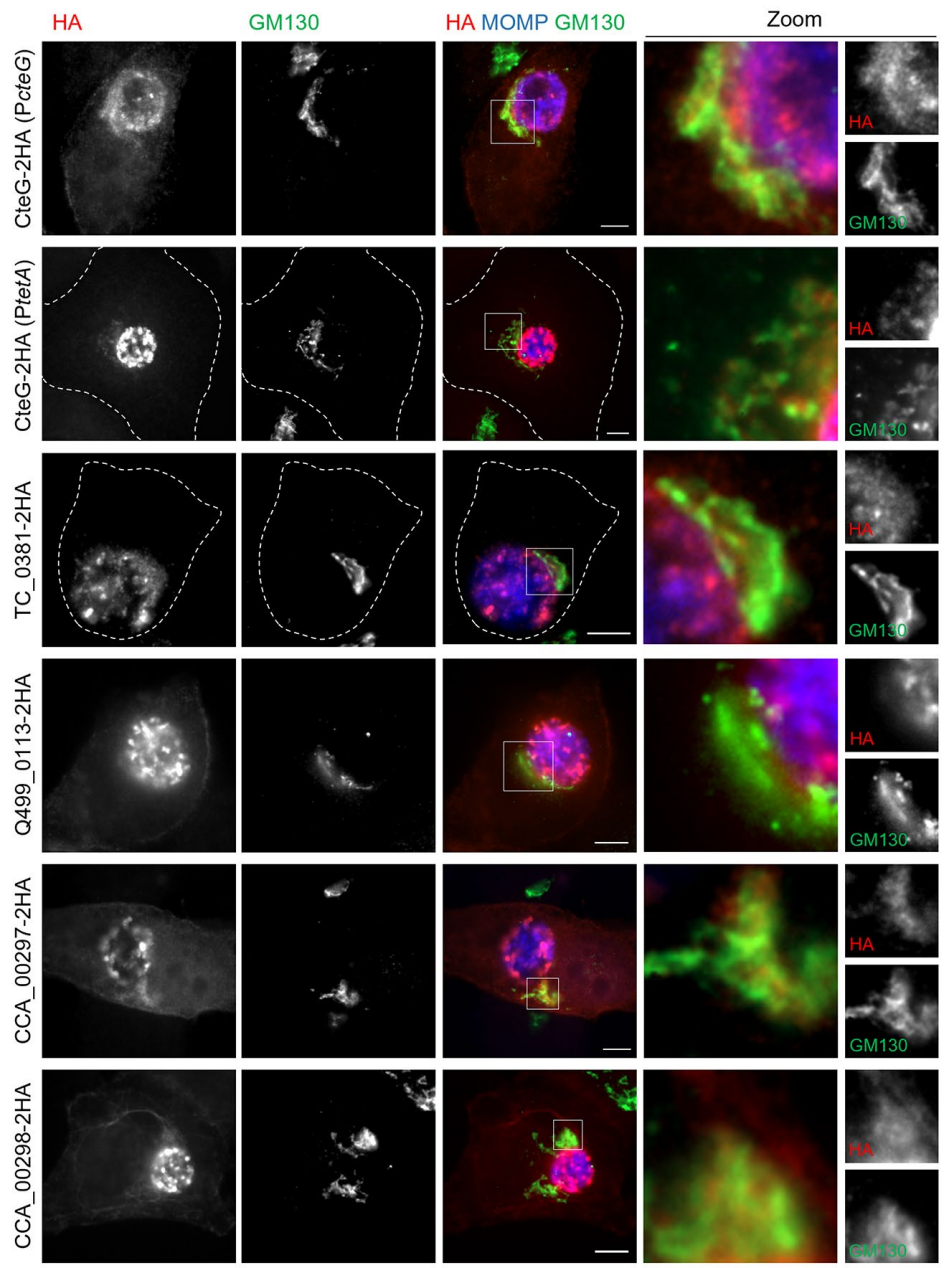
Subcellular localization of CteG homologs in Chlamydiaceae delivered by *C. trachomatis* into host cells infected for 24 h. HeLa cells were infected for 24 h with *C. trachomatis cteG::aadA* harboring plasmids encoding CteG or CteG homologs within Chlamydiaceae (Q499_0133 from *C. suis*; CAB376 from *C. abortus*; CCA00389, CCA00297 and CCA00298, from *C. caviae*; G5S_0733 from *C. pecorum*; TC_0381, from *C. muridarum*) with a 2HA C-terminal epitope tag. The gene encoding CteG was expressed from its own promoter or from the *tetA* promoter (as indicated) and the genes encoding its homologs within Chlamydiaceae were mostly expressed from the *tetA* promoter, except for the gene encoding TC_0381 that was expressed from the *cteG* promoter. Infected cells were fixed with 4% (w/v) paraformaldehyde and immunolabelled with antibodies against HA (red), *cis*-Golgi protein GM130 (green) and *C. trachomatis* Major Outer Membrane Protein (MOMP; blue), and appropriate fluorophore-conjugated secondary antibodies. The immunolabeled cells were analysed by immunofluorescence microscopy. The indicated regions of overlap between the GM130/Golgi and HA/chlamydial protein immunofluorescence signals were magnified and are shown as zoomed images. Scale bars, 5 μm

**Fig. 8:**
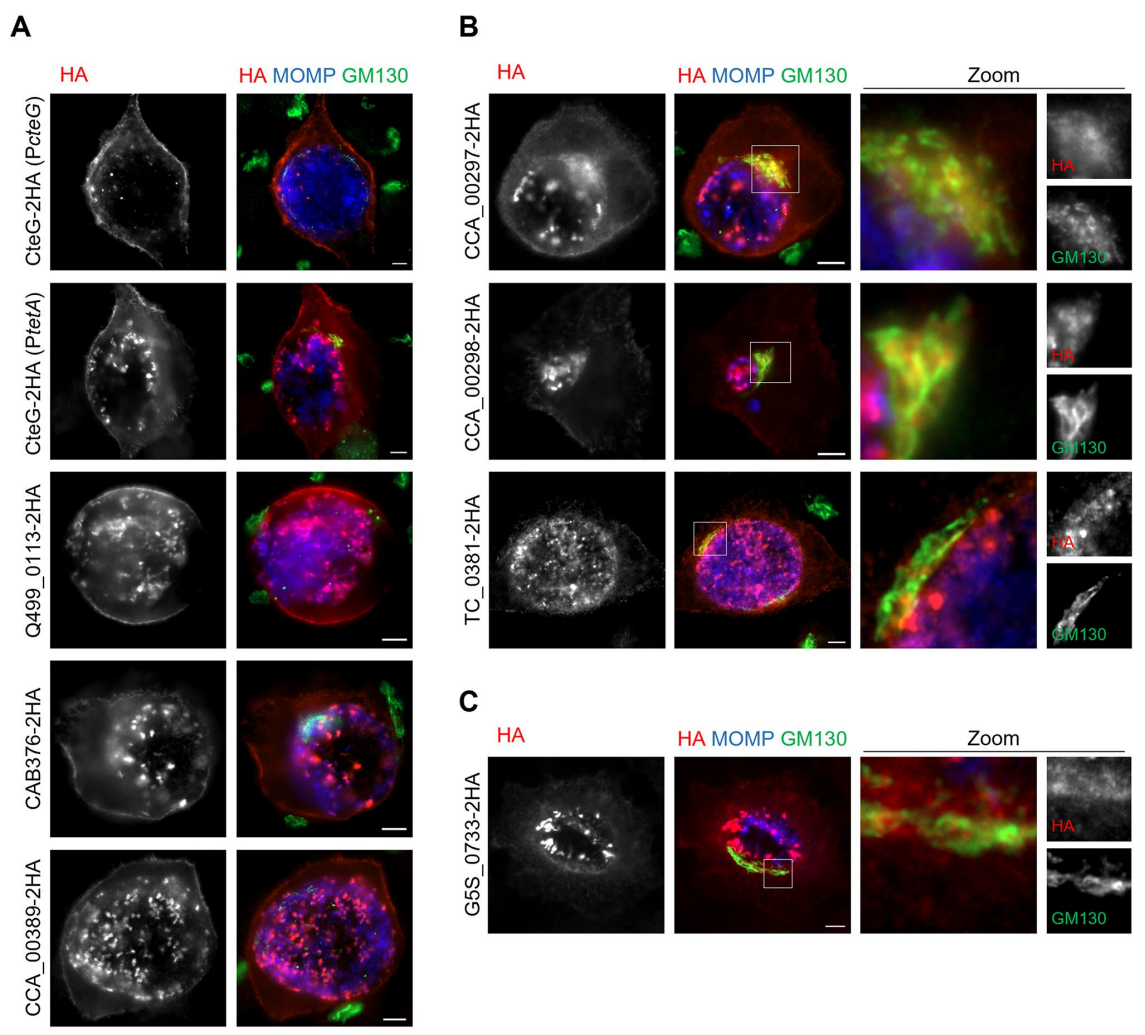
Subcellular localization of CteG homologs in Chlamydiaceae delivered by *C. trachomatis* into host cells infected for 46 h. HeLa cells were infected for 46 h, as detailed in the legend of Fig. 7. The images illustrate localization of CteG and its homologs at the cell periphery **(A)**, at the cell periphery and Golgi region **(B)**, or solely at the Golgi region **(C)**. In **(B)** and **(C)**, the indicated regions of overlap between the GM130/Golgi and HA/chlamydial protein immunofluorescence signals were magnified and are shown as zoomed images

To compare the subcellular localization of the CteG homologs that were delivered into HeLa cells by *C. trachomatis* to the known localization of CteG at the Golgi (at about 24 h p.i.) and at the plasma membrane (at late infection times such as 46 h p.i.), HeLa cells infected for 24 and 46 h were immunolabelled with antibodies against HA, *C. trachomatis* MOMP, and the *cis*-Golgi protein GM130, followed by fluorophore-conjugated secondary antibodies. Analysis by fluorescence microscopy of cells infected for 24 h showed that CteG homologs that were delivered by *C. trachomatis* into host cells also localized at the Golgi region (*C. muridarum* TC_0381, *C. suis* Q499_0113, *C. caviae* CCA_00297, and *C. caviae* CCA_00298; Fig. 7) and partially at the cell periphery (*C. suis* Q499_0113, *caviae* CCA_00297, and *C. caviae* CCA_00298; Fig. 7 and Fig. S6), which is suggestive of a plasma membrane localization. At 46 h p.i., several CteG homologs clearly localized at the cell periphery (*C. suis* Q499_0113, *C. caviae* and CCA_00389, and *C. abortus* CAB376; Fig. 8A), at the Golgi region and partially at the cell periphery (*C. muridarum* TC_0381, *C. caviae* CCA_00297, and *C. caviae* CCA_00298; Fig. 8B), or only at the Golgi region (*C. pecorum* G5S_0733; Fig. 8C). The data on the subcellular localization of CteG homologs is summarized in Table 1.

In summary, and in general, the experiments analysing secretion of CteG homologs by *Y. enterocolitica* and their delivery into host cells by *C. trachomatis* indicate that they are also T3S substrates, and many can be delivered into host cells by *C. trachomatis* and localize at the Golgi region and periphery of infected cells.

## Discussion

In this work, we revealed that CteG shows a unique expansion within Chlamydiaceae, characterized by a total of at least 62 homologs, including several inparalogs and outparalogs in various *Chlamydia* and *Chlamydiifrater* species. Phylogenetically, the homologs of *C. trachomatis* CteG defined two clades that correlate with synteny of their encoding genes to *C. trachomatis cteG* (clade CteG I) or synteny between the encoding genes but not with *C. trachomatis cteG* (clade CteG II). In contrast, regardless of being CteG I or CteG II, almost all CteG homologs tested were type III secreted by *Y. enterocolitica*. Furthermore, most of the homologs that were type III secreted were also delivered by *C. trachomatis* into host cells where they localized at the Golgi region and/or at the cell periphery, suggesting a plasma membrane localization. Future studies should clarify if the identified homologs are functionally resemblant to *C. trachomatis* CteG regarding its known capacities to promote lytic exit from host cells [22] and to induce centrosome amplification [23], or even its putative ability to interfere with eukaryotic vesicular trafficking [17]. Interestingly, *C. trachomatis* CteG may have additional functions early in the chlamydial infectious cycle as *cteG* gene expression peaks between 1 and 2 h p.i. [17, 35, 55].

Based on the distribution, phylogeny and synteny analyses, a duplication event probably took place in an ancestor of *Chlamydia* species that originated two *cteG*-related paralogous genes in two separate loci. This is supported by the presence of CteG homologs from clade CteG II in *Chlamydiifrater* species. One of the paralogs was eventually lost in *C. muridarum*, *C. trachomatis*, and *C. suis* (CteG II, loci non-sytenic to *cteG*) and the other paralog in *C. avium* and *C. gallinacea* (CteG I, loci syntenic to *cteG*). There is also evidence for more recent within locus duplication events as several *Chlamydia* species show more than one *cteG*-related gene within a single locus (e.g. *C. pecorum* and *Chlamydiifrater*).

Gene duplication is a major mechanism in the evolution of eukaryotes [56], but it is thought to be less common in prokaryotes, where horizontal gene transfer has a more important role in driving protein diversification [57–59]. There are, however, examples of gene duplication in bacterial genomes [60, 61]. In general, if there is no selective advantage in maintaining a duplicated gene, then the gene is inactivated by mutation and is eventually deleted from the genome. When the duplicated gene is maintained, this is normally associated to a novel function (neofunctionalization), segregation of the functions of the ancestral gene (subfunctionalization), or to conservation of functions in both duplicates to provide redundancy and robustness to environmental challenges or if increased gene dosage is favourable [56]. At present, when considering the multiple functions (host cell lytic exit and centrosome duplication) described for *C. trachomatis* CteG [22, 23], as well as possible additional functions earlier in the infectious cycle [17, 35, 55], and the wide diversity of hosts of the Chlamydiaceae [43], all hypothetical scenarios that normally explain the maintenance of duplicated genes seem possible to explain the apparent importance of effective gene duplication in the evolution of CteG homologs.

There are two families of effectors that show paralogs in *C. trachomatis*: the DUF582 proteins (CT619, CT620, CT621, C711, and CT712) [15], which are transported to the host cell nucleus [15, 62] and target the eukaryotic ESCRT (endosomal sorting complexes required for transport) machinery [63], and the deubiquitinases Cdu1 and Cdu2 [42], which have been shown to limit the host response [64], to mediate Golgi fragmentation [65], and to control chlamydial exit by stabilizing other effectors [66]. It should be noted that in our preliminary tBLASTx search for putative homologs of different *C. trachomatis* effectors, the paralogs of CT619 in *C. trachomatis* were not detected, despite having been so in other *Chlamydia* and *Chlamydiifrater* species. Regardless of this lack of robustness of the initial reciprocal tBLASTx approach, it is very unlikely that the expansion of *C. trachomatis* effectors other than CteG within Chlamydiaceae would have not been detected. Differently from CteG, the duplication events that likely led to the five or four paralogs of DUF582 proteins present in Chlamydiaceae species probably occurred earlier in evolution, as they are present in all *Chlamydia* and *Chlamydiifrater* species. As for the deubiquitinases, it is known that there is a significant expansion of ubiquitination-related gene families in Chlamydiae [67]. However, homologs of Cdu1 are present in many, but not all, Chlamydiaceae [66], and the presence of both Cdu1 and Cdu2 is exclusive of *C. trachomatis*, *C. muridarum* and *C. suis* [66]. Overall, this suggests a recent duplication event that led to the emergence of Cdu2, and not a generalized expansion in Chlamydiaceae as observed for CteG.

Most CteG homologs are type III secreted by *Y. enterocolitica* and are delivered into host cells by *C. trachomatis*. The signals that direct proteins for T3S are normally located in their first 20–30 amino acids [68], and this seems to be the case of CteG [35]. Therefore, it could be puzzling that most *C. trachomatis* CteG homologs do not show significant sequence similarity in their N-terminal regions. However, while the exact nature of the T3S signal is still elusive, the amino sequence of the T3S signal can tolerate multiple changes, and a flexible and non-structured N-terminus region may play an important role in targeting substrates to the T3S machinery [69]. All this may explain why CteG homologs are almost all secreted by *Yersinia* and many delivered into host cells by *C. trachomatis* despite the lack of sequence similarity in their N-termini. [17]Not considering the cases of *C. suis* Q499-0114 and *C. pecorum* G5S_0729, whose annotated open-reading frames were disrupted in the strains used to amplify their gene DNA, for the putative homologs of CteG that were not type III secreted by *Y. enterocolitica* (*C. pecorum* G5S_0731) and/or delivered into host cells by *C. trachomatis* (*C. pneumoniae* Cpn_0404 and Cpn_0405), they may have evolved as cytosolic proteins of chlamydiae. Alternatively, their secretion by *Y. enterocolitica* and/or delivery into host cells by *C. trachomatis* could require specific targeting signals or uncharacterized endogenous factors, such as T3S chaperones [70], hypothetically present in the original species but not in *Y. enterocolitica* and/or *C. trachomatis*.

We previously showed that the first 100 amino acids residues of CteG function as a Golgi targeting region after ectopic expression in uninfected cells [17], However, the exact Golgi and plasma membrane targeting signals of *C. trachomatis* CteG in infected cells are presently unknown. As discussed above for the T3S signal, there is no significant sequence similarity in the N-terminal region of CteG (including the described Golgi targeting region; [17]) with its homologs and only modest similarity over the entire polypeptide sequence. Despite this, when CteG homologs are delivered by *C. trachomatis* into host cells, generally, they are also directed to the Golgi region and to the cell periphery of infected cells. This suggests that the Golgi and plasma membrane targeting signals of CteG should be conserved among its homologs, and further supports that they should also be effectors.

For most *C. trachomatis* effectors, the paralogy detected by the tBLASTx approach in *C. trachomatis* (i.e., no paralogs, or presence of one or more paralogous pairs) is either maintained across Chlamydiaceae species or putative homologs are only found in a few Chlamydiaceae species. This is particularly striking for the Incs analysed, with some seemingly only present in *C. trachomatis* and *C. suis* (CT222, CT224, CT225), in *C. trachomatis* and *C. muridarum* (CT227), or in *C. trachomatis*, *C. suis* and *C. muridarum* (IncA, IncD-G, CT134, CT135, CT192, CT226, CT228, CpoS, CT249, CT345, and InaC), while most of the others appear to be present in all (IncS, IncB, CT440, CT565, and CT850) or almost all Chlamydiaceae species (IncV, CT006, MrcA, IncC, IncM, CT383, CT442, CT483, and CT618). This contrasts with a previous study analysing the distribution of Incs in Chlamydiaceae that led to the identification of 23 “core Incs” present in all *Chlamydia* species [21]. However, the genomic sequences available at the time were limited to *C. trachomatis*, *C. muridarum*, *C. caviae*, and *C. pneumoniae*. From our analysis, such “core Incs” are limited to IncV, CT006, MrcA, IncS, IncB, CT383, CT440, CT442, CT565, and CT850, when considering those that have been experimentally detected in the inclusion membrane.

In conclusion, we showed that CteG homologs are present in all Chlamydiaceae and their evolution was likely driven by gene duplication, which is uncommon in bacteria. Despite modest sequence conservation, most CteG homologs are delivered into host cells where they localize in the Golgi region and cell periphery, thus suggesting that they are also effectors. Future additional functional studies on the CteG effector family may provide important insights on the molecular functions of *C. trachomatis* CteG, on how bacterial effector proteins evolved, and on the relevance of gene duplication in the evolution of obligate intracellular bacterial pathogens.

## Electronic supplementary material

Below is the link to the electronic supplementary material.

Supplementary Material 1

Supplementary Material 2

Supplementary Material 3

## Data Availability

Data is provided within the manuscript or supplementary information files. Additionally, original tree files and alignments can be found in Figshare: https://figshare.com/s/b5ee1bbacc3d9ec347fb.
