# Supplementary material for: Identification of homologs of the Chlamydia trachomatis effector CteG reveals a family of Chlamydiaceae type III secreted proteins that can be delivered into host cells
Source: Med Microbiol Immunol. 2024 Jul 15;213(1):15. doi: 10.1007/s00430-024-00798-9 (PMC11249467; doi:10.1007/s00430-024-00798-9)
Supplement: Supplementary file 2 — Supplementary Material 2 [file 430_2024_798_MOESM2_ESM.pdf]

SUPPLEMENTAL MATERIAL

Identification of homologs of the *Chlamydia trachomatis* effector CteG reveals a family of Chlamydiaceae type III secreted proteins that can be delivered into host cells

Inês Serrano Pereira[1,2], Maria da Cunha[1,2], Inês Leal[1,2], Maria Pequito Luís[1,2], Paula Gonçalves[1,2], Carla Gonçalves[1,2], Luís Jaime Mota[1,2#]

[1]Associate Laboratory i4HB - Institute for Health and Bioeconomy, NOVA School of Science and Technology, NOVA University Lisbon, Caparica, Portugal.

[2]UCIBIO – Applied Molecular Biosciences Unit, Department of Life Sciences, NOVA School of Science and Technology, NOVA University Lisbon, Caparica, Portugal.

[#]Address correspondence to Luís Jaime Mota, ljmota@fct.unl.pt

Table S1. Plasmids used in this study.

Plasmid	Characteristics and construction	Source/Ref.
pLJM3	Expresses YopE under the control of its own promoter (P_{yopE}).	[1]
pFA61	Derivative of pLJM3. Expresses RplJ-HA under the control of P_{yopE}.	[2]
pRM7	Derivative of pLJM3. Expresses CT105/CteG-HA under the control of P_{yopE}.	[2]
pMM1	Derivative of pLJM3. Expresses *C. pneumoniae* Cpn0404-HA under the control of P_{yopE}. A DNA fragment containing Cpn_0404 was amplified from genomic DNA of *C. pneumoniae* using primers 2366 and 2367. The resulting DNA product was digested with NdeI and HindIII and inserted into those sites of pLJM3.	This work.
pMM2	Derivative of pLJM3. Expresses *C. pneumoniae* Cpn0405-HA under the control of P_{yopE}. A DNA fragment containing Cpn_0405 was amplified from genomic DNA of *C. pneumoniae* using primers 2368 and 2369. The resulting DNA product was digested with NdeI and HindIII and inserted into those sites of pLJM3.	This work.
pMM3	Derivative of pLJM3. Expresses *C. muridarum* TC_0381-HA under the control of P_{yopE}. A DNA fragment containing TC_0381 was amplified from genomic DNA of *C. muridarum* using primers 2370 and 2371. The resulting DNA product was digested with NdeI and XhoI and inserted into those sites of pLJM3.	This work.
pMC103	Derivative of pLJM3. Expresses *C. suis* Q499_0113-HA under the control of P_{yopE}. Because Q499_0113 contains one NdeI site in its sequence, two DNA products were amplified from genomic DNA of *C. suis* S45/6 using primer pairs 2374 and 2376, and 2377 and 2375. The two DNA products were then fused by overlapping PCR using primers 2374 and 2375. The resulting DNA product (without the internal NdeI site) was digested with NdeI and XhoI and inserted into those sites in pLJM3.	This work.
pMC104	Derivative of pLJM3. Expresses C. suis Q499_0114A-HA under the control of P_{yopE}. A DNA fragment containing Q499_0114A was amplified from genomic DNA of *C. suis* S45/6 using primers 2372 and 2785. The resulting DNA product was digested with NdeI and HindIII and inserted into those sites of pLJM3.	This work

Table S1. Plasmids used in this study. (*Continued*).

Plasmid	Characteristics and construction	Source/Ref.
pMC105	Derivative of pLJM3. Expresses *C. abortus* CAB376-HA under the control of P_{yopE}. A DNA fragment containing CAB376 was amplified from genomic DNA of *C. abortus* ADN-A87 using primers 2726 and 2707. The resulting DNA product was digested with NdeI and XhoI and inserted into those sites of pLJM3.	This work.
pMC106	Derivative of pLJM3. Expresses *C. caviae* CCA_00389-HA under the control of P_{yopE}. A DNA fragment containing CCA_00389 was amplified from genomic DNA of *C. caviae* GPIC using primers 2727 and 2708. The resulting DNA product was digested with NdeI and XhoI and inserted into those sites of pLJM3.	This work.
pMC107	Derivative of pLJM3. Expresses *C. caviae* CCA_00390-HA under the control of P_{yopE}. Because CCA_00390 contains one NdeI site in its sequence, two DNA products were amplified from genomic DNA of *C. caviae* GPIC using primer pairs 2728 and 2330, and 2729 and 2709. The two DNA products were then fused by overlapping PCR using primers 2728 and 2709. The resulting DNA product (without the internal NdeI site) was digested with NdeI and KpnI and inserted into those sites in pLJM3.	This work.
pMC108	Derivative of pLJM3. Expresses *C. caviae* CCA_00297-HA under the control of P_{yopE}. Because CCA_00297 contains one NdeI site in its sequence, two DNA products were amplified from genomic DNA of *C. caviae* GPIC using primer pairs 2731 and 2716, 2732 and 2710. The two DNA products were then fused by overlapping PCR using primers 2731 and 2710. The resulting DNA product (without the internal NdeI site) was digested with NdeI and KpnI and inserted into those sites in pLJM3.	This work.
pMC109	Derivative of pLJM3. Expresses *C. caviae* CCA_00298-HA under the control of P_{yopE}. A DNA fragment containing CCA_00298 was amplified from genomic DNA of *C. caviae* GPIC using primers 2717 and 2711. The resulting DNA product was digested with NdeI and XhoI and inserted into those sites of pLJM3.	This work.

Table S1. Plasmids used in this study. (*Continued*).

Plasmid	Characteristics and construction	Source/Ref.
pMC110	Derivative of pLJM3. Expresses *C. pecorum* G5S_0729-HA under the control of P_{yopE}. A DNA fragment containing G5S_0729 was amplified from genomic DNA of *C. pecorum* E58 using primers 2718 and 2772. The resulting DNA product was digested with NdeI and XhoI and inserted into those sites of pLJM3.	This work.
pMC111	Derivative of pLJM3. Expresses *C. pecorum* G5S_0731-HA under the control of P_{yopE}. Because G5S_0731 contains two NdeI sites in its sequence, three DNA products were amplified from genomic DNA of *C. pecorum* E58 using primer pairs: 2719 and 2721, 2720 and 2723, and 2722 and 2784. The three DNA products were then fused by a two-step overlapping PCR using primers 2719 and 2723 (first) followed by a final PCR with primers 2719 and 2784. The resulting DNA product (without the two internal NdeI sites) was digested with NdeI and KpnI and inserted into those sites in pLJM3.	This work.
pMC112	Derivative of pLJM3. Expresses *C. pecorum* G5S_0733-HA under the control of P_{yopE}. A DNA fragment containing G5S_0733 was amplified from genomic DNA of *C. pecorum* E58 using primers 2724 and 2714. The resulting DNA product was digested with NdeI and KpnI and inserted into those sites of pLJM3.	This work.
pMC113	Derivative of pLJM3. Expresses *C. suis* Q499_0114B under the control of P*yopE*. A DNA fragment containing Q499_0114B was amplified from genomic DNA of *C. suis* S45/6 using primers 2777 and 2786. The resulting DNA product was digested with NdeI and XhoI and inserted into those sites of pLJM3.	This work.
pSVP247	Derivative of p2TK2—SW2 for expression of proteins in *C. trachomatis* with a C-terminal double 2HA tag. Contains the terminator of the *incDEFG* operon of *C. trachomatis* L2/434.	[3]
pSVP264/pCteG-2HA	Derivative of pSVP247 for the expression of CteG-2HA under the control of the predicted *cteG* promoter (*PcteG*).	[3]
pSVP302/pTet-CteG-2HA	Derivative of pSVP247 encoding CteG-2HA under the control of the P_{tetA}.	[3]

Table S1. Plasmids used in this study. (*Continued*).

Plasmid	Characteristics and construction	Source/Ref.
pMC114	Derivative of pSVP247 with the tetracycline-inducible promoter (P_{tetA}). P_{tetA} was amplified from pSVP302 using primers 2792 and 2793. The resulting DNA product was digested with KpnI and NdeI and inserted into those sites of pSVP247.	This work.
pMC115	Derivative of pMC114. Encodes *C. suis* Q499_0113-2HA under the control of the P_{tetA}. A DNA fragment containing Q499_0113 was amplified from pMC103 using primers 2374 and 2794. The resulting DNA product was digested with NdeI and NotI and inserted into those sites of pMC114.	This work.
pMC116	Derivative of pMC114. Encodes *C. suis* Q499_0114A-2HA under the control of the P_{tetA}. A DNA fragment containing Q499_0114A was amplified from pMC104 using primers 2372 and 2795. The resulting DNA product was digested with NdeI and NotI and inserted into those sites of pMC114.	This work.
pMC117	Derivative of pMC114. Encodes *C. pneumoniae* Cpn0404-2HA under the control of the P_{tetA}. A DNA fragment containing Cpn_0404 was amplified from pMM1 using primers 28 and 2808. The resulting DNA product was digested with NdeI and NotI and inserted into those sites of pMC114.	This work.
pMC118	Derivative of pMC114. Encodes *C. pneumoniae* Cpn0405-2HA under the control of the P_{tetA}. A DNA fragment containing Cpn_0405 was amplified from pMM2 using primers 2368 and 2796. The resulting DNA product was digested with NdeI and NotI and inserted into those sites of pMC114.	This work.
pMC119	Derivative of pMC114. Encodes *C. muridarum* TC_0381-2HA under the control of the P_{tetA}. A DNA fragment containing TC_0381 was amplified from pMM3 using primers 2370 and 2797. The resulting DNA product was digested with NdeI and NotI and inserted into those sites of pMC114.	This work.
pMC120	Derivative of pMC114. Encodes *C. abortus* CAB376-2HA under the control of the P_{tetA}. A DNA fragment containing CAB376 was amplified from pMC105 using primers 2726 and 2798. The resulting DNA product was digested with NdeI and NotI and inserted into those sites of pMC114.	This work.

Table S1. Plasmids used in this study. (*Continued*).

Plasmid	Characteristics and construction	Source/Ref.
pMC121	Derivative of pMC114. Encodes *C. caviae* CCA_00389-2HA under the control of the P_{tetA}. A DNA fragment containing CCA_00389 was amplified from pMC106 using primers 2727 and 2799. The resulting DNA product was digested with NdeI and NotI and inserted into those sites of pMC114	This work.
pMC122	Derivative of pMC114. Encodes *C. caviae* CCA_00390-2HA under the control of the P_{tetA}. A DNA fragment containing CCA_00390 was amplified from pMC107 using primers 2728 and 2800. The resulting DNA product was digested with NdeI and NotI and inserted into those sites of pMC114.	This work.
pMC123	Derivative of pMC114. Encodes *C. caviae* CCA_00297-2HA under the control of the P_{tetA}. A DNA fragment containing CCA_00297 was amplified from pMC108 using primers 2731 and 2801. The resulting DNA product was digested with NdeI and NotI and inserted into those sites of pMC114.	This work.
pMC124	Derivative of pMC114. Encodes *C. caviae* CCA_00298-2HA under the control of the P_{tetA}. A DNA fragment containing CCA_00298 was amplified from pMC109 using primers 2717 and 2802. The resulting DNA product was digested with NdeI and NotI and inserted into those sites of pMC114	This work.
pMC125	Derivative of pMC114. Encodes *C. pecorum* G5S_0729-2HA under the control of the P_{tetA}. A DNA fragment containing G5S_0729 was amplified from pMC110 using primers 2718 and 2803. The resulting DNA product was digested with NdeI and NotI and inserted into those sites of pMC114.	This work.
pMC126	Derivative of pMC114. Encodes *C. pecorum* G5S_0733-2HA under the control of the P_{tetA}. A DNA fragment containing G5S_0733 was amplified from pMC112 using primers 2724 and 2804. The resulting DNA product was digested with NdeI and NotI and inserted into those sites of pMC114.	This work.

Table S1. Plasmids used in this study. (*Continued*).

Plasmid	Characteristics and construction	Source/Ref.
pIL1	Derivative of pSVP247. Encodes Q499_0114A-2HA under the control of the *PcteG*. A DNA fragment containing Q499_0114A was amplified from pMC116 using primers 2836 and 2795, and a DNA fragment containing the *cteG* promoter was amplified from pSVP264 using primers 2837 and 2838. After overlapping PCR with primers 2837 and 2795, the resulting DNA product was digested with NdeI and NotI and inserted into those sites of pSVP247.	This work.
pIL3	Derivative of pSVP247. Encodes TC_0381-2HA under the control of the *PcteG*. A DNA fragment containing TC_0381 was amplified from pMC119 using primers 2797 and 2841, and a DNA fragment containing *cteG* promoter was amplified from pSVP264 using primers 1680 and 2842. The resulting DNA product was digested with KpnI and NotI and inserted into those sites of pSVP247.	This work.

Table S2. DNA primers used in this study.

Number	Description	Sequence (5' → 3')
28	pLJM3 sequencing forward primer; also used to construct pMC117.	GATTAAGTTGGGTAACGCC
29	pLJM3 sequencing reverse primer.	TTGTGTGGAATTGTGAGCG
1680	Forward primer used to construct pIL3.	GATCGGTACCTTCTTTATTATTGAGAAACG
2366	Cpn0404_NdeI_F; forward primer used to construct pMM1.	GATCTTTCATATGAGCTTATTATCAGGACATCG
2367	Cpn0404_HindIII_R; reverse primer used to construct pMM1.	GATCAAGCTTAAGCATAATCAGGAACATCATACGGATAAAACATAACCTCCTCTTCTTC
2368	Cpn0405_NdeI_F; forward primer used to construct pMM2 and pMC118.	GATCTTTCATATGGGTTTCACTGATTACTTAGG
2369	Cpn0405_HindIII_R; reverse primer used to construct pMM2.	GATCAAGCTTAAGCATAATCAGGAACATCATACGGATATAAAGGGATTGCGGGTTCTTGGG
2370	TC_0381_NdeI_F; forward primer used to construct pMM3 and pMC119.	GATCTTTCATATGTCGTTAGGTATTAGTGGC
2371	TC_0381_XhoI_R; reverse primer used to construct pMM3	GATCCTCGAGTTAAGCATAATCAGGAACATCATACGGATAAGCCTCAGTAACCTTTACCAC
2372	Q499_0114_F_NdeI; forward primer used to construct pMC104 and pMC116.	GATCTTTCATATGTCTGCACTAGGTAGCATTTG
2374	Q499_0113_F_NdeI; forward primer used to construct pMC103 and pMC115.	GATCTTTCATATGTCATCTGTTATTAATGGG
2375	Q499_0113_R_XhoI; reverse primer used to construct pMC103.	GATCCTCGAGTTAAGCATAATCAGGAACATCATACGGATAGTTGCCGGTGGAGACAACTCC
2376	Q499_0113_Rv_NdeI_mut; used to construct pMC103 – by overlapping PCR creates silent mutation that inactivates NdeI site.	CAGTCGGTTGGGTACAGATGAGAGAAGGCGAAG
2377	Q499_0113_Fwd_NdeI_mut; used to construct pMC103 – by overlapping PCR creates silent mutation that inactivates NdeI site.	CTTCGCCTTCTCTCATCTGTACCCAACCGACTG

Table S2. DNA primers used in this study. (*Continued*).

Number	Description	Sequence (5' → 3')
2707	CAB376_XhoI_R; reverse primer used to construct pMC105.	GATCCTCGAGTTAAGCATAAT<br>CAGGAACATCATACGGATACA<br>CTGAGGTCTCGCGATACTC
2708	CCA_00389_XhoI_R; reverse primer used to construct pMC106.	GATCCTCGAGTTAAGCATAAT<br>CAGGAACATCATACGGATACA<br>CGGCAGTTTCTCTATAC
2709	CCA_00390_KpnI_R; reverse primer used to construct pMC107.	GATCGGTACCTTAAGCATAAT<br>CAGGAACATCATACGGATAAA<br>CTATAGTTTCTTCTTCC
2710	CCA_00297_KpnI_R; reverse primer used to construct pMC108.	GATCGGTACCTTAAGCATAAT<br>CAGGAACATCATACGGATATC<br>TTAAAAATAGACTCGAAGTC
2711	CCA_00298_XhoI_R; reverse primer used to construct pMC109.	CCGCTCGAGTTAAGCATAATC<br>AGGAACATCATACGGATATTC<br>CCCATACTCTTGTCGC
2714	G5S_0733_KpnI_R; reverse primer used to construct pMC112.	GATCGGTACCTCAAGCATAAT<br>CAGGAACATCATACGGATAAC<br>AATCTTCTTCATCAAAAGTTTG
2716	CCA_00297_NdeI_mut_R; used to construct pMC108 – by overlapping PCR creates silent mutation that inactivates NdeI site.	AATTGCTCTAAGCAGATGGGG<br>AGTTCTTG
2717	CCA_00298_NdeI_F; forward primer used to construct pMC109 and pMC124.	GATCTTTCATATGTGTTTCCCA<br>GGTTGTCCCAA
2718	G5S_0729_NdeI_F; forward primer used to construct pMC110 and pMC125.	GATCTTTCATATGACAACACC<br>AACAAGTTCTATAGC
2719	G5S_0731_NdeI_F; forward primer used to construct pMC111.	GATCTTTCATATGGAATTGGG<br>GGAGGGGGGG
2720	G5S_0731_NdeI_mut1_F; used to construct pMC111 – by overlapping PCR creates silent mutation that inactivates NdeI site.	AGCTTTGTCCATAGCCTATGTC<br>ATTTCCTCTG
2721	G5S_0731_NdeI_mut1_R; used to construct pMC111 – by overlapping PCR creates silent mutation that inactivates NdeI site.	CAGAGGAAATGACATAGGCTA<br>TGGACAAAGCT
2722	G5S_0731_NdeI_mut2_F; used to construct pMC111 – by overlapping PCR creates silent mutation that inactivates NdeI site.	GTCTTTATGGCTTCACATGCAT<br>CAGCTTTTAAGC
2723	G5S_0731_NdeI_mut2_R; used to construct pMC111 – by overlapping PCR creates silent mutation that inactivates NdeI site.	GCTTAAAAGCTGATGCATGTG<br>AAGCCATAAAGAC

Table S2. DNA primers used in this study. (*Continued*).

Number	Description	Sequence (5' → 3')
2724	G5S_0733_NdeI_F; forward primer used to construct pMC112 and pMC126.	GATCTTTCATATGACGAATCCTACAGGTCGGGG
2726	CAB376_NdeI_F; forward primer used to construct pMC105 and pMC120.	GATCTTTCATATGACATTTCCATTAGGGAATATACG
2727	CCA_00389_NdeI_F; forward primer used to construct pMC106 and pMC121.	GATCTTTCATATGGCATTTCCATTAGGGAATACG
2728	CCA_00390_NdeI_F; forward primer used to construct pMC107 and pMC122.	GATCTTTCATATGTGGTTATTATACGCTCAC
2729	CCA_00390_NdeI_mut_F; used to construct pMC107 – by overlapping PCR creates silent mutation that inactivates NdeI site.	TTAAGGGCTCCTACACACATGCCAATTAGTG
2730	CCA_00390_NdeI_mut_R; used to construct pMC107 – by overlapping PCR creates silent mutation that inactivates NdeI site.	CACTAATTGGCATGTGTGTAGGAGCCCTTAA
2731	CCA_00297_ NdeI_F; forward primer used to construct pMC108 and pMC123.	GATCTTTCATATGTGCTGCCCTGGAATTAGCGC
2732	CCA_00297_NdeI_mut_F; used to construct pMC108 – by overlapping PCR creates silent mutation that inactivates NdeI site.	CAAGAACTCCCCATCTGCTTAGAGCAATT
2760	CAB376iseq; sequencing primer.	AAATGAGACAGTCGTGTTACG
2762	CCA_00389i seq; sequencing primer.	AGTGGAGGTAGAGTTAAGGC
2763	CCA_00390 iseq1; sequencing primer.	TGATTCTGTTGTTACTACGC
2764	CCA_00390 iseq2; sequencing primer.	AATCCCTGGAAGATCTCTAG
2767	Q499_0114i seq 1200F; sequencing primer.	CTGTGAGCTTGAGTCTCTGC
2768	Q499_0114i seq 740R; sequencing primer.	TCTCTTATCAGGACAACAACC
2769	Q499_0114i seq 600F; sequencing primer.	AGTTCAACATTCTGTACAC
2770	Q499_0113i seq 700R; sequencing primer.	TGGCTAAGTAATAGAGATGCC

Table S2. DNA primers used in this study. (*Continued*).

Number	Description	Sequence (5' → 3')
2771	Q499_0113i seq 1400F; sequencing primer.	TTCTGGAAACAGTCCGTTC
2772	G5S_0729A_ XhoI_R (new); reverse primer used to construct pMC112.	GATCCTCGAGCTAAGCATAATCAGGAACATCATACGGATAATGCCTTGATATGCATCCTTTCC
2777	Q499_0114B_NdeI_F; forward primer used to construct pMC113.	GATCCTTCATATGAATTCTTATACAGTACATCTATTCG
2784	G5S_0731_KpnI_R; reverse primer used to construct pMC111.	GATCGGTACCTTAAGCATAATCAGGAACATCATACGGATAAGATATCTCTGAGTTGTCTTGC
2785	Q499_0114A_XhoI_R; reverse primer used to construct pMC104.	GATCCTCGAGTTAAGCATAATCAGGAACATCATACGGATAGCCCAGCCAAGGGTACCCAAAAGG
2786	Q499_0114B_XhoI_R; reverse primer used to construct pMC113.	GATCCTCGAGTTAAGCATAATCAGGAACATCATACGGATAGCAAGTAGCTTCTCTCTG
2792	pTET_NdeI_R; reverse primer used to construct pMC114.	GATCCATATGTTCACTTTTCTCTATCACTGATAGG
2793	pTETKpn_mut_NdeI_F; forward primer used to construct pMC114.	GATCGGTACCTTAAGACCCACTTTCACATTTAAGTTGTTTTTCTAATCCGCAGATGATCAATTCAAGGCC
2794	Q499_0113_NotI_R; reverse primer used to construct pMC115.	GATCGCGGCCGCGATAGTTGCCGGTGGAGACAACTCC
2795	Q499_0114A_NotI_R; reverse primer used to construct pMC116 and pIL1.	GATCGCGGCCGCGGCCCAGCCAAGGGTACCC
2796	Cpn0405_NotI_R; reverse primer used to construct pMC118.	GATCGCGGCCGCGTAAAGGGATTGCGGGTTCTTGG
2797	TC0381_NotI_R; reverse primer used to construct pMC119 and pIL3.	GATCGCGGCCGCGAGCCTCAGTAACCTTTACCAC
2798	CAB376_NotI_R; reverse primer used to construct pMC120.	GATCGCGGCCGCGCACTGAGGTCTCGCGATAC
2799	CCA_00389_NotI_R; reverse primer used to construct pMC121.	GATCGCGGCCGCGCACGGCAGTTTCTCTATACTCC

Table S2. DNA primers used in this study. (*Continued*).

Number	Description	Sequence (5’ → 3’)
2800	CCA_00390_NotI_R; reverse primer used to construct pMC122.	GATCGCGGCCGCGAACTATAG TTTCTTCTTCCTCTTC
2801	CCA_00297_NotI_R; reverse primer used to construct pMC123.	GATCGCGGCCGCGTCTTAAAA ATAGACTCGAAGTC
2802	CCA_00298_NotI_R; reverse primer used to construct pMC124.	GATCGCGGCCGCGTTCCCCAT ACTCTTGTCGC
2803	G5S_0729_NotI_R; reverse primer used to construct pMC125.	GATCGCGGCCGCGATGCCTTG ATATGCATCCTTTCC
2804	G5S_0733_NotI_R; reverse primer used to construct pMC126.	GATCGCGGCCGCGACAATCTT CTTCATCAAAAGTTTG
2808	Cpn0404 NotI_R; reverse primer used to construct pMC117.	GATCGCGGCCGCGAAACATAA CCTCCTCTTCTTC
2836	Used for overlapping PCR to construct pIL1.	GAATCCGGGAGTTAAAGGTAT GTCTGCACTAGGTAGC
2837	Forward primer used to construct pIL1.	GATCCATATGGGTACCTTCTTT ATTATTGAGAAACG
2838	Used for overlapping PCR to construct pIL1.	GCTACCTAGTGCAGACATACC TTTAACTCCCGGATTC
2841	Used for overlapping PCR to construct pIL3.	CCACTAATACCTAACGACATA CCTTTAACTCCCGGATTC
2842	Used for overlapping PCR to construct pIL3.	GAATCCGGGAGTTAAAGGTAT GTCGTTAGGTATTAGTGG

Table S3. *C. trachomatis* strains used and constructed in this study.

Strains	Description	Source/Refs.
cteG::aadA	Derivative of wild-type strain L2/434/Bu ACE05 with *cteG* inactivated.	[3]
cteG::aadA (pSVP264)	Derivative of *cteG::aadA* carrying pSVP264/pCteG-2HA (P_{cteG}).	[3]
cteG::aadA (pSVP302)	Derivative of *cteG::aadA* carrying pSVP302/pCteG-2HA(P_{tetA}).	This work.
cteG::aadA (pMC115)	Derivative of *cteG::aadA* carrying pMC120/Q499_0113-2HA (P_{tetA}).	This work.
cteG::aadA (pMC116)	Derivative of *cteG::aadA* carrying pMC116/Q499_0114A-2HA (P_{tetA}).	This work.
cteG::aadA (pMC117)	Derivative of *cteG::aadA* carrying pMC117/Cpn_0404-2HA (P_{tetA}).	This work.
cteG::aadA (pMC118)	Derivative of *cteG::aadA* carrying pMC118/Cpn_0405-2HA (P_{tetA}).	This work.
cteG::aadA (pMC119)	Derivative of *cteG::aadA* carrying pMC119/TC_0381-2HA (P_{tetA}).	This work.
cteG::aadA (pMC120)	Derivative of *cteG::aadA* carrying pMC120/CAB376-2HA (P_{tetA}).	This work.
cteG::aadA (pMC121)	Derivative of *cteG::aadA* carrying pMC121/CCA_00389-2HA (P_{tetA}).	This work.
cteG::aadA (pMC123)	Derivative of *cteG::aadA* carrying pMC123/CCA_00297-2HA (P_{tetA}).	This work.
cteG::aadA (pMC124)	Derivative of *cteG::aadA* carrying pMC124/CCA_00298-2HA (P_{tetA}).	This work.
cteG::aadA (pMC125)	Derivative of *cteG::aadA* carrying pMC125/G5S_0729-2HA (P_{tetA}).	This work.
cteG::aadA (pMC126)	Derivative of *cteG::aadA* carrying pMC126/G5S_0733-2HA (P_{tetA}).	This work.
cteG::aadA (pIL1)	Derivative of *cteG::aadA* carrying pIL1/Q499_0114A-2HA (P_{cteG}).	This work.
cteG::aadA (pIL3)	Derivative of *cteG::aadA* carrying pIL3/pTC_0381-2HA (P_{cteG}).	This work.

Table S4. Putative homologues of *C. trachomatis* effector proteins in other *Chlamydia* and *Chlamydiifrater* species.

(See attached Excel file).

Table S5. CteG putative homologs within Chlamydiaceae whose sequences were used to generate the phylogeny of CteG.

(See attached Excel file).

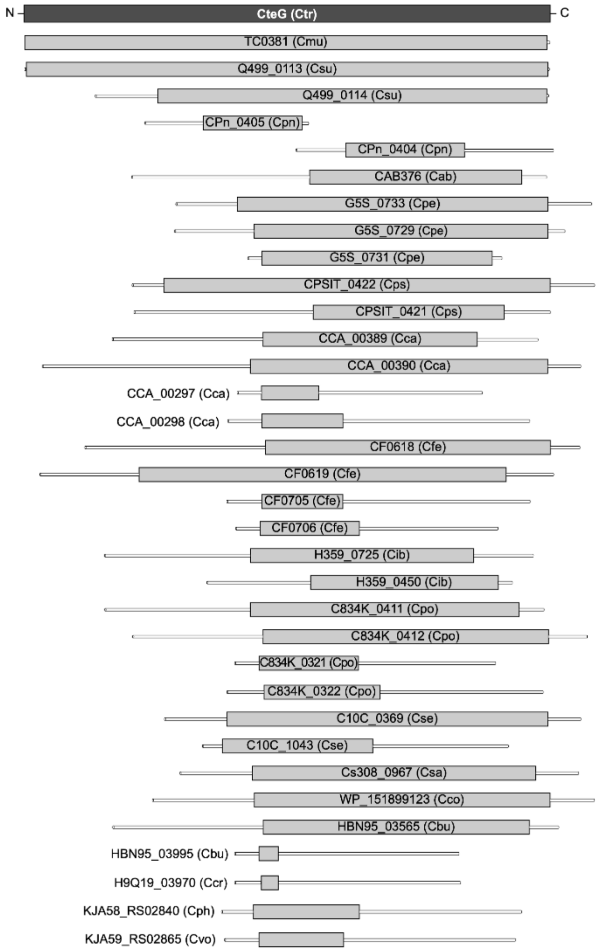

	Protein (aa)	aa range CteG	aa range homolog	e-value	Id.
CteG (Ctr)	**656**				
TC0381 (Cmu)	650	1-651	1-644	0.0	53%
Q499_0113 (Csu)	650	4-654	7-648	7e-170	47%
Q499_0114 (Csu)	607	177-651	141-603	3e-44	28%
CPn_0405 (Cpn)	258	233-350	132-245	8e-10	30%
CPn_0404 (Cpn)	339	401-555	12-165	7e-07	22%
CAB376 (Cab)	732	361-612	400-683	2e-14	28%
G5S_0733 (Cpe)	594	279-654	138-509	3e-25	28%
G5S_0729 (Cpe)	567	288-653	179-534	5e-20	25%
G5S_0731 (Cpe)	342	294-587	32-323	3e-15	25%
CPSIT_0422 (Cps)	610	189-656	72-523	2e-26	25%
CPSIT_0421 (Cps)	733	366-597	403-643	2e-13	27%
CCA_00389 (Cca)	726	297-570	339-607	4e-20	27%
CCA_00390 (Cca)	898	282-651	467-833	3e-17	26%
CCA_00297 (Cca)	445	290-370	55-125	0.003	27%
CCA_00298 (Cca)	533	294-397	77-168	0.003	24%
CF0618 (Cfe)	816	301-656	406-758	2e-12	24%
CF0619 (Cfe)	737	143-601	223-644	2e-19	26%
CF0705 (Cfe)	536	294-397	79-170	2e-05	25%
CF0706 (Cfe)	447	290-422	55-176	9e-04	23%
H359_0725 (Cib)	720	283-563	328-603	1e-13	27%
H359_0450 (Cib)	488	363-593	233-460	1e-05	21%
C834K_0411 (Cpo)	730	284-612	327-681	5e-21	27%
C834K_0412 (Cpo)	721	298-653	293-645	5e-18	24%
C834K_0321 (Cpo)	444	290-422	55-176	0.013	23%
C834K_0322 (Cpo)	526	300-444	84-208	3e-06	23%
C10C_0369 (Cse)	599	266-651	140-534	1e-25	26%
C10C_1043 (Cse)	461	256-431	45-196	0.007	20%
Cs308_0967 (Csa)	589	286-642	163-505	7e-27	24%
WP_151899123 (Cco)	672	288-655	229-585	7e-18	22%
HBN95_03565 (Cbu)	736	297-636	338-678	2e-25	25%
HBN95_03995 (Cbu)	441	290-323	55-88	0.008	38%
H9Q19_03970 (Ccr)	444	295-323	60-80	0.009	38%
KJA58_RS02840 (Cph)	524	288-429	72-207	1e-14	31%
KJA59_RS02865 (Cvo)	519	290-397	78-183	1e-16	40%

Figure S1. Amino acid sequence alignment between *C. trachomatis* CteG and its putative homologs within Chlamydiaceae. The amino acid sequence of CteG from *C. trachomatis* strain L2/434 and of its putative homologs within Chlamydiaceae as identified by the reciprocal tBLASTx approach (Fig. 1) were individually aligned using BLASTp. The region of each CteG putative homolog that shows identity to CteG is drawn and illustrated within a grey rectangle (on scale). The extent of the N- and C-terminal regions of each CteG putative homolog that does not display significant identity to CteG is also depicted as thin white rectangles (not on scale). The following information is also indicated for each case: total protein length in amino acids (aa); range of amino acids within CteG that displays significant identity to CteG homologs (aa range CteG); range of amino acids within CteG putative homologs that displays significant identity to CteG (aa range target); e-value obtained in the BLASTp (e-value); percentage of identity between the amino acid sequence of CteG and of its putative homologs (Id.); *C. trachomatis* (Ctr); *C. muridarum* (Cmu); *C. suis* (Csu); *C. pneumoniae* (Cpn); *C. abortus* (Cab); *C. pecorum* (Cpe); *C. caviae* (Cca); *C. felis* (Cfe); *C. ibidis* (Cib); *C. poikilothermis* (Cpo); *C. serpentis* (Cse); *C. sanzinia* (Csa); *C. corallus* (Cco); *C. psittaci* (Cps); *C. buteonis* (Cbu); *C. crocodili* (Ccr); *C. volucris* (Cvo); *C. phoenicopteri* (Cph).

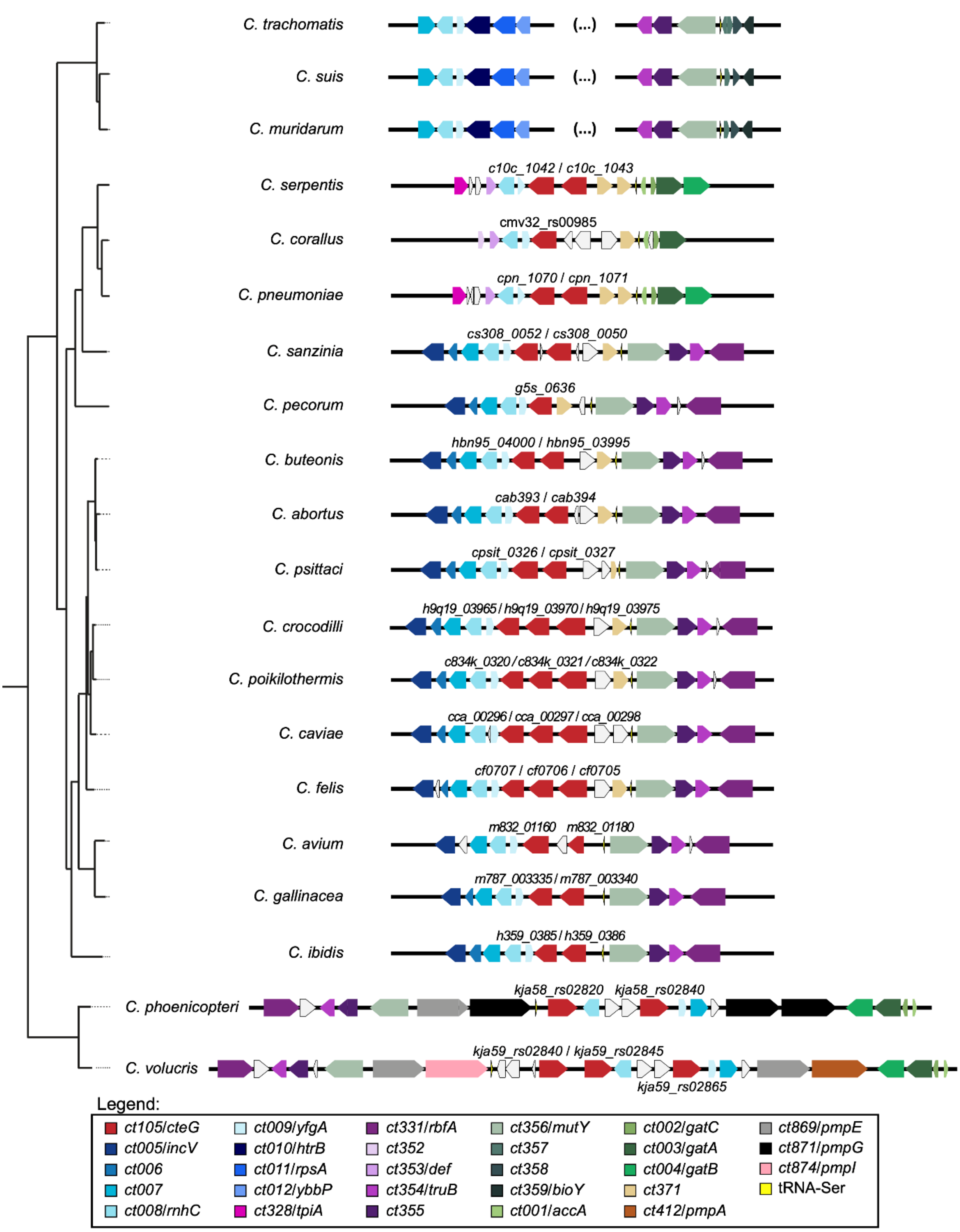

Figure S2. Genomic region in *Chlamydia* and *Chlamydiifrater* species of *cteG* homologs that are non-syntenic to *C. trachomatis cteG*. *cteG* homologs identified by the analysis of CteG phylogeny depicted in Fig. 3 are coloured in red (see also Table S5). Other genes are coloured as indicated within the figure. Genes for which no putative homologs were found are coloured in white. Genomic regions are depicted according to the species tree in Fig. 2. The nomenclature in the legend for each group of putative homologs is from *C. trachomatis* D/UW3 strain [4].

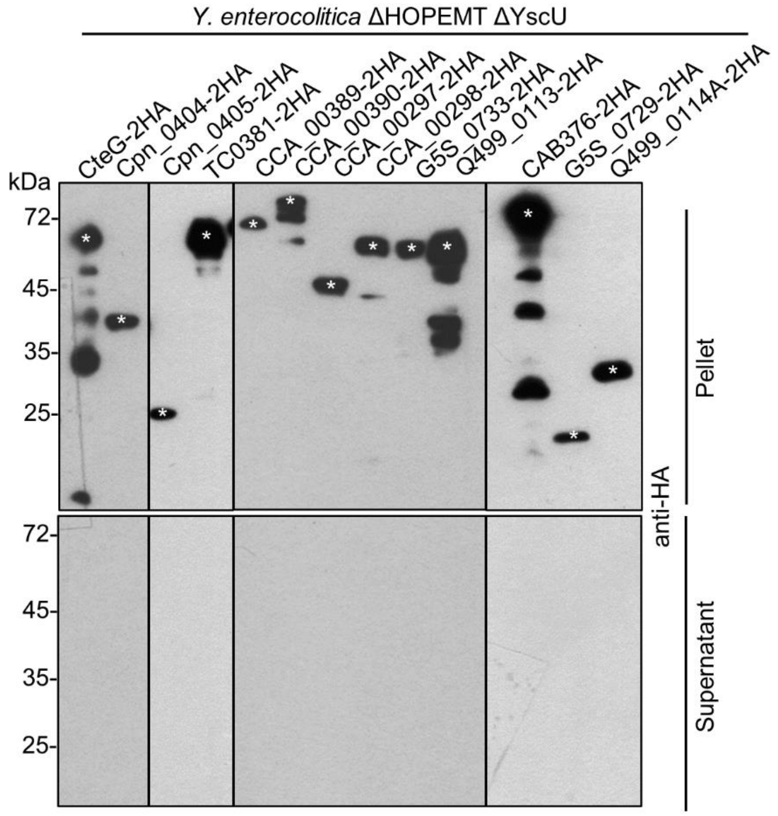

Figure S3. Analysis of type III secretion (T3S) of CteG homologs within Chlamydiaceae using *Y. enterocolitica* ΔHOPEMT ΔYscU. *Y. enterocolitica* ΔHOPEMT ΔYscU is a T3S-deficient isogenic derivative of T3S-proficient *Y. enterocolitica* ΔHOPEMT and was used to confirm T3S-dependency of the secretion by the ΔHOPEMT strain of identified CteG homologs within Chlamydiaceae with a C-terminal HA epitope tag. Immunoblots show the result of T3S assays in which proteins in culture supernatants (S, secreted proteins) and in bacterial pellets (P, non-secreted proteins) from ~5 x 10^8 and ~5 x 10^7 bacteria, respectively, were loaded per lane. CteG is a known *C. trachomatis* T3S substrate [2], RplJ is a *C. trachomatis* ribosomal protein that is not type III secreted. The band corresponding to the predicted molecular mass of CteG or of CteG homolog proteins is indicated with a white asterisk.

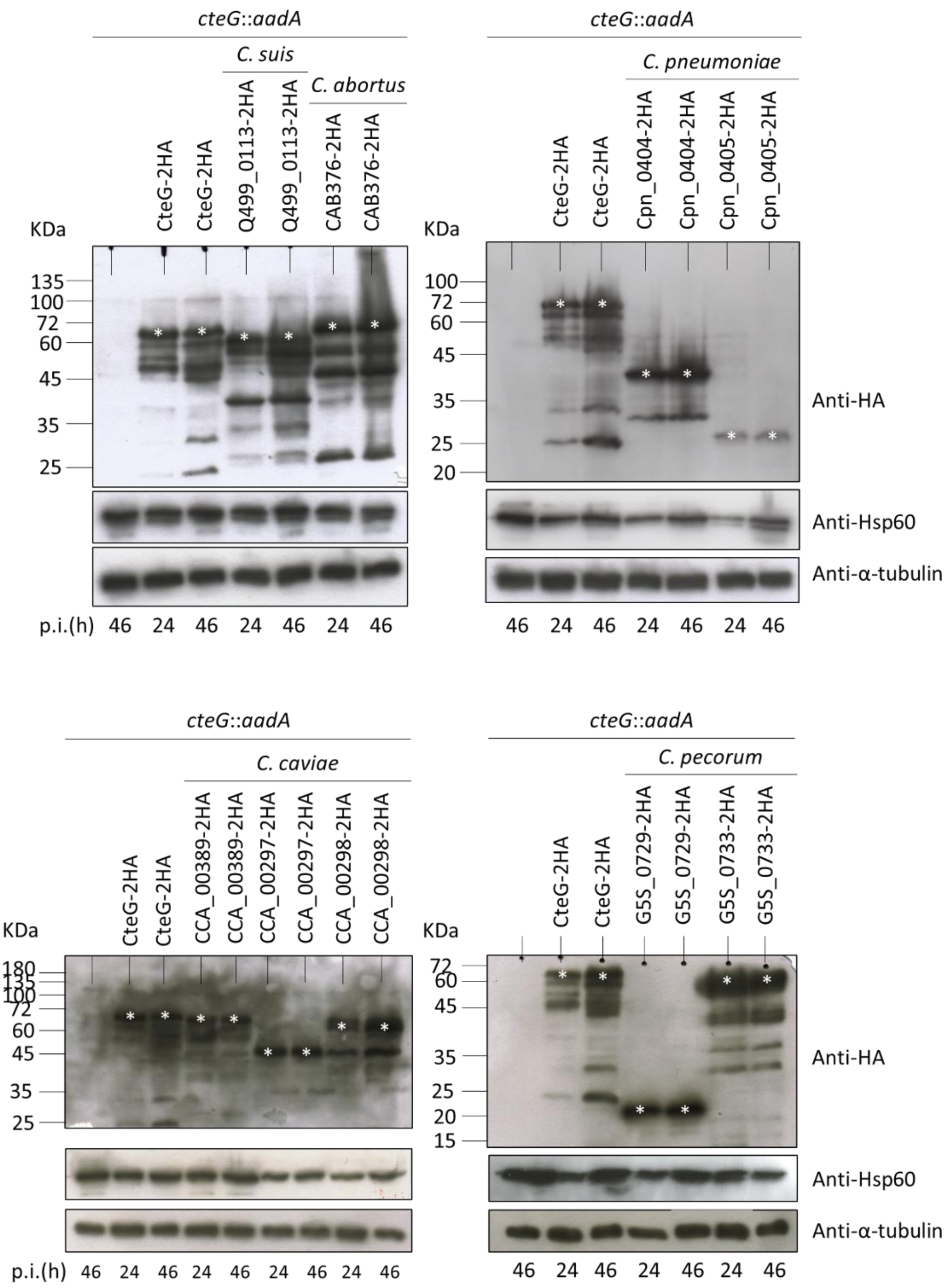

Figure S4. Immunoblotting analysis of the production of CteG homologs after expression of their encoding genes by the *tetA* promoter. HeLa 229 cells were infected with *C. trachomatis cteG::aadA* harbouring pP_{tet}-CteG-2HA, pP_{tet}-Q499_0113-2HA, pP_{tet}-Q499_0114A-2HA, pP_{tet}-TC_0381-2HA, pP_{tet}-CAB376-2HA, pP_{tet}-Cpn_0404-2HA, pP_{tet}-Cpn_0405-2HA, pP_{tet}-CCA_00389-2HA, pP_{tet}-CCA_00297-2HA, pP_{tet}-CCA_00298-2HA, pP_{tet}-G5S_0729-2HA or pP_{tet}-G5S_0733-2HA. At 24 and 46 h p.i., whole cell extracts were prepared and then analysed by immunoblotting with antibodies against HA, *C. trachomatis* Hsp60 (bacterial loading control) and human α-tubulin (HeLa cell loading control). The detection was made using SuperSignal West Pico detection kit (Thermo Fisher Scientific) to detect Hsp60 or α-tubulin, or SuperSignal West Femto detection kit (Thermo Fisher Scientific) to detect 2HA-tagged proteins. The band corresponding to the predicted molecular mass of CteG or of CteG homolog proteins is indicated with a white asterisk.

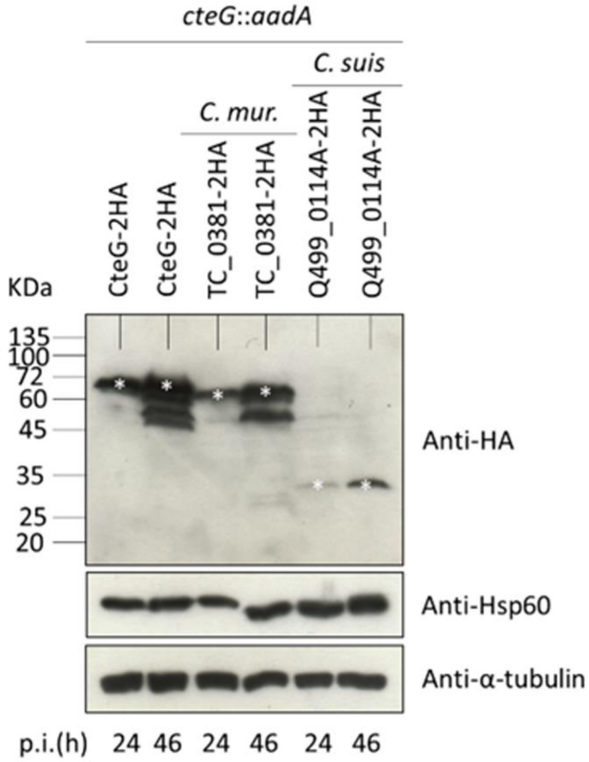

Figure S5. Immunoblotting analysis of the production of CteG homologs after expression of their encoding genes from the *cteG* promoter. HeLa cells were infected with *C. trachomatis cteG::aadA* harbouring pP_{cteG}-CteG-2HA, pP_{cteG}-TC_0381-2HA or pP_{cteG}-Q499_0114A-2HA. At 24 and 46 h p.i., whole cell extracts were prepared and then analysed as described in the legend of Fig. S4. The band corresponding to the predicted molecular mass of CteG or CteG homolog proteins is indicated with a white asterisk.

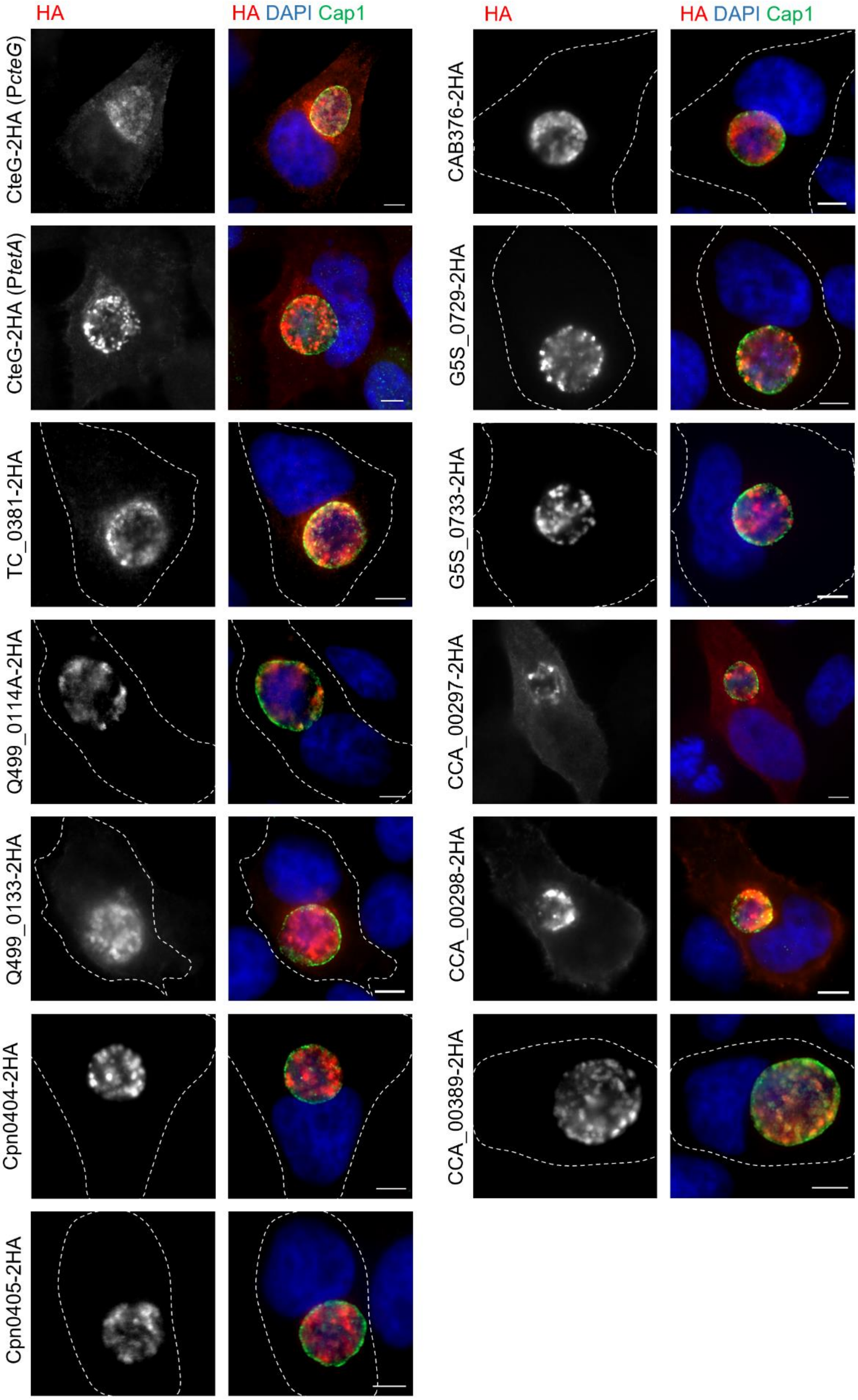

Figure S6. Delivery into host cells by *C. trachomatis* of CteG homologs in Chlamydiaceae. HeLa cells were infected for 24 h with *C. trachomatis cteG::aadA* harboring plasmids encoding CteG or CteG homologs within Chlamydiaceae (Q499_0133 and Q499_0114A, from *C. suis*; Cpn0404 and Cpn0405,

from *C. pneumoniae*; CAB376 from *C. abortus*; CCA00389, CCA00297 and CCA00298, from *C. caviae*; G5S_0733 and G5S_0729, from *C. pecorum*; TC_0381, from *C. muridarum*) with a 2HA C-terminal epitope tag. The gene encoding CteG was expressed from its own promoter (P*cteG*) or from the *tetA* promoter (P*tet*) and the genes encoding its homologs within Chlamydiaceae were mostly expressed from P*tet*, except for the genes encoding TC_0381 and Q499_0114A that were expressed from P*cteG*. Infected cells were fixed with 4% (w/v) paraformaldehyde and immunolabelled with antibodies against HA (red) and the inclusion membrane-localized protein Cap1 (green), and appropriate fluorophore-conjugated secondary antibodies. The host and chlamydial DNA were stained with DAPI (blue). The immunolabeled and stained cells were analysed by fluorescence microscopy. Scale bars, 5 µm. See also Fig. 6.

Supplementary materials references (all cited in main text)

1. Marenne MN, Journet L, Mota LJ, Cornelis GR (2003) Genetic analysis of the formation of the Ysc-Yop translocation pore in macrophages by *Yersinia enterocolitica*: role of LcrV, YscF and YopN. Microb Pathog. 35:243-58. https://doi:10.1016/s0882-4010(03)00154-2.
2. da Cunha M, Milho C, Almeida F, Pais SV, Borges V, Mauricio R, Borrego MJ, Gomes JP, Mota LJ (2014) Identification of type III secretion substrates of *Chlamydia trachomatis* using *Yersinia enterocolitica* as a heterologous system. BMC Microbiol. 14:40. https://doi:10.1186/1471-2180-14-40.
3. Pais SV, Key CE, Borges V, Pereira IS, Gomes JP, Fisher DJ, Mota LJ (2019) CteG is a *Chlamydia trachomatis* effector protein that associates with the Golgi complex of infected host cells. Sci Rep. 9:6133. https://doi:10.1038/s41598-019-42647-3.
4. Stephens RS, Kalman S, Lammel C, Fan J, Marathe R, Aravind L, Mitchell W, et al. (1998) Genome sequence of an obligate intracellular pathogen of humans: *Chlamydia trachomatis*. Science. 282:754-9. https://doi:10.1126/science.282.5389.754.
